# Evolution of the ribosomal exit tunnel through the eyes of the nascent chain

**DOI:** 10.1093/nar/gkag689

**Published:** 2026-07-07

**Authors:** Tomasz Włodarski

**Affiliations:** Department of Bioinformatics, Institute of Biochemistry and Biophysics, Polish Academy of Sciences, Warsaw 02-106, Poland

## Abstract

The exit tunnel is a universally conserved feature of the ribosome that directs the nascent polypeptide into the cellular environment and is involved in co-translational folding, stalling, and antibiotic binding. While cryogenic electron microscopy has revealed variations in ribosome structure, tunnel definition and comparative analyses have largely relied on geometric algorithms. Here, we present a functional, nascent chain (NC)-centric characterization of the exit tunnel across the tree of life, derived from molecular dynamics simulations of 64 cytoplasmic ribosome structures. By mapping steric accessibility through the “eyes” of the NC at five distinct stages of translation, we reveal a topological and stage-dependent complexity invisible to geometric approaches, demonstrating how tunnel accessibility dynamically changes during biosynthesis. We identify transient, bacteria-specific lateral branches that, in archaeal and eukaryotic ribosomes, are structurally occluded by the eL39 protein and N-terminal extensions of the uL24 protein. These evolutionary “plugs” seal the tunnel wall and decrease its functional width. Collectively, our results demonstrate that the ribosome exit tunnel has a branched, lineage-specific topology where accessibility is temporally gated by NC length. This functional definition provides a new framework for understanding how ribosomal architecture modulates the early stages of protein biogenesis.

## Graphical Abstract

**Graphical Abstract:**
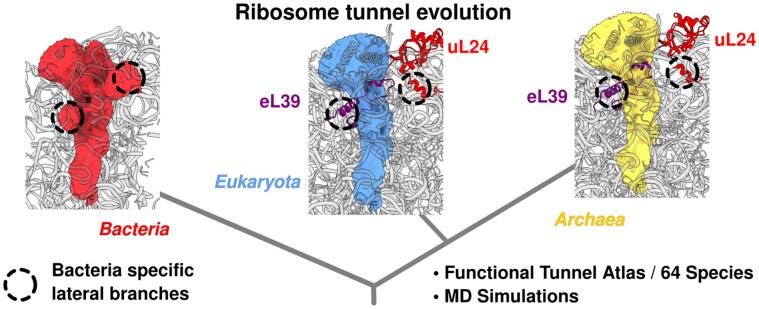

## Introduction

The ribosome is an ancient biomolecular machine responsible for translating genetic information into proteins in all living systems [1]. After peptide bond formation in the peptidyl transferase centre (PTC) [2], the nascent polypeptide exits the ribosome through a narrow channel—the ribosomal exit tunnel. This structural feature is conserved across all domains of life [1]; remarkably, every protein ever produced by life on Earth has passed through it.

The tunnel, including its vestibule, is universally constructed from ribosomal RNA and conserved ribosomal proteins, including uL4, uL22, uL23, uL24, and uL29. Eukaryotes and archaea also incorporate the specific protein eL39, which replaces the uL23 exit tunnel loop. This tunnel provides the first opportunity for the nascent chain (NC) to fold; however, due to its narrow diameter, only α-helices and small β-hairpins can form within it [3–9]. More complex structures fold in or beyond the vestibule as part of the co-translational folding process [7, 10–18]. Modifications to the exit tunnel can directly influence the co-translational folding, either through rational engineering of tunnel loops [19, 20] or by using ribosomal protein paralogs, such as RPL39L [21]. Additionally, the tunnel is a regulatory hotspot: it interacts with specific NCs to modulate elongation and induce translational stalling [22–25], and it is a well-established target for ribosome-binding antibiotics [26, 27].

Historically, the existence of the tunnel was inferred from proteolytic experiments [28], confirmed with antibody binding [29], and finally visualized by electron microscopy [30]. Earlier low-resolution cryogenic electron microscopy (cryo-EM) studies suggested the presence of possible side branches and alternative exits [31]. However, the first in-depth analysis of the ribosome tunnel, conducted using the *Haloarcula marismortui* ribosome with a rolling ball algorithm [32], found no tunnel branching and confirmed a narrow geometry that precludes substantial folding.

Subsequent molecular dynamics (MD) simulations further characterized the physicochemical complexity of the tunnel using the *H. marismortui* ribosome. The 3D potentials of mean force (PMFs) calculated for five representative amino acids showed that distinct residues experience different energetic landscapes throughout the ribosomal exit tunnel [33]. As each 3D PMF differed significantly, this suggests the tunnel is sensitive to the chemical nature of the NC. Mapping the electrostatic potential has also shown that the tunnel is highly negatively charged and heterogeneous [34], which can modulate elongation speed [35, 36]. Furthermore, water dynamics within the tunnel differ significantly from bulk water [37], potentially weakening the hydrophobic effect in the vestibule region [38], highlighting the complexity of this confined environment.

A comparative study of 20 distinct ribosome structures across all domains of life recently revealed that tunnel geometry broadly mirrors phylogenetic relationships and identified a previously unrecognized “second constriction” site in eukaryotes [39]. It also found that the upper part of the tunnel, closer to the PTC, is generally more structurally conserved than the lower part, which includes the vestibule. Subsequently, it has been proposed that the resulting narrower eukaryotic tunnel could act as a kinetic trap for small proteins [40]. Most recently, this geometric classification has been extended and refined to show that certain eukaryotic lineages, such as parasitic protists, can revert to wider, bacteria-like tunnel architectures [41]. Additionally, we recently demonstrated that the tunnel vestibule may support at least two main NC pathways, with a preference that depends on folding status and translation stage [42].

Despite these significant insights, most previous studies have focused on overall geometry (radius and volume) rather than on how the NC experiences the tunnel. Consequently, while broad phylogenetic variations in tunnel geometry are established [39], a major outstanding question remains: how does tunnel accessibility for the NC change dynamically during biosynthesis, and how do lineage-specific adaptations functionally reshape this environment? To address this, we adopt a functional approach to explore the structural heterogeneity of ribosome tunnels across various species by analysing them through the “eyes” of the NC. To map the shape of the tunnel, we followed all-atom MD trajectories of the NC and converted them into 3D density maps that reflect the spatial occupancy of the NC within the tunnel. Moreover, we generated these maps for different stages of translation, based on the hypothesis that the local accessibility of the tunnel changes during biosynthesis.

We leverage the recent increase in cryo-EM ribosome structures, which has more than tripled the number of unique high-resolution cytoplasmic ribosomes from 18 to 64 [39]. Our structural analysis confirms the previously identified geometric constriction sites but reveals a far more complex topology defined by transient accessibility. We characterized a third constriction site defined by eukaryotic/archeal-specific eL39 protein and identified additional bifurcation tunnels (“lateral branches”) observed exclusively in bacteria, which are gated by NC length. Interestingly, in eukaryotes and archaea, these branches are closed by alternative conformations of conserved proteins or ribosomal RNA (rRNA), specifically through the incorporation of the eL39 protein and N-terminal extension of uL24. These additional tunnel branches are only accessible to the NC of certain lengths and provide a compelling representation of how the tunnel architecture is experienced differently by the NC at early stages of translation. Using this functional approach, we provide another argument that the tunnel cannot be accurately described as a simple linear cylinder with a varying radius, as its topology is far more complex. Finally, we mapped the changes in the physicochemical environment along the tunnel pathway, providing a rich resource for future studies of NC-tunnel interactions and the discovery of potential drug-binding sites.

## Materials and methods

### Search strategy for unique ribosome structures

To comprehensively collect known ribosome structures from the Protein Data Bank (PDB) [43], we used a dual-strategy approach that combined keyword-based and sequence-similarity searches. First, we performed a search using the term “ribosome” in the Structure Keywords field, refining the results to include only structures containing both protein and RNA components determined by either X-ray crystallography or single-particle cryo-electron microscopy. Second, as an orthogonal approach, we selected from the PDB all experimentally determined structures that contained at least one protein chain with ≥30% sequence identity to any known highly conserved uL2 protein. These two approaches yielded 2620 and 2111 PDB entries, respectively, resulting in a total of 2830 unique ribosome structures. As a final validation step, we queried the Ribosome Analysis Database (https://radtool.rc.northeastern.edu/) to confirm that no structures were omitted from our analysis. From this dataset, we selected a single representative structure per organism, prioritizing high resolution and the presence of NC (translating ribosomes), while strictly excluding structures containing antibiotics or other factors bound in or near the ribosomal exit tunnel. For example, the available structures for *Cutibacterium acnes* were excluded due to the presence of tunnel-bound sarecycline (Supplementary Table S5). Our final dataset comprised 64 unique cytoplasmic ribosome structures, of which 20 were from Bacteria (Supplementary Table S1), 38 from Eukaryotes (Supplementary Table S2), and 6 from Archaea (Supplementary Table S3).

### Definition of the reference ribosome tunnel

To build the reference model of the ribosome exit tunnel, we used our previously published *Escherichia coli* ribosome structure containing the FLN5 filamin domain NC (PDB ID: 7zp8) [19]. Initially, we selected all ribosomal residues within 30 Å of the available NC model using VMD [44] to define the preliminary tunnel boundaries. To capture the full extent of the ribosomal tunnel accessible to the NC, we used all-atom structure-based MD simulations. The NC was modelled as a poly-alanine chain with an N-terminal formylmethionine (N-fMet) at five distinct lengths (L = 10, 20, 30, 40, and 60 amino acids). These lengths were chosen to represent various stages of translation; the L = 40 chain was sufficient to span the entire length of the tunnel, whereas the L = 60 chain allowed sampling of the ribosome surface surrounding the exit tunnel. All-atom structure-based models (SBMs) were generated using SMOG2 (template: SBM_AA-amber-bonds) [45–47] for these five ribosome NC complexes (RNCs). For each system, 5 × 10^7^ time steps of simulations were conducted using GROMACS 2021 [48] (see details below), with ribosomal atoms and the C-terminal residue of the NC held fixed. In SBM, these time steps correspond to reduced units rather than absolute physical time; a detailed discussion on the position-dependent calibration of effective simulated timescales can be found in [49]. We mapped the resulting MD trajectories onto the full *E. coli* ribosome structure (PDB ID: 7zp8) [19] and, using a 6 Å cut-off, identified additional tunnel residues that were missed in the initial selection. This approach provided a more comprehensive definition of the reference ribosome tunnel.

### Structural alignment and selection of the ribosome exit tunnels

The reference ribosome exit tunnel from *E. coli* was used to guide the selection of the exit tunnels from the remaining 63 ribosome structures. Given the complexity of aligning whole ribosome structures across diverse organisms, we leveraged available cryo-EM maps to facilitate the process. We initially performed a rigid-body fit of each target ribosome structure into the cryo-EM density map of the reference *E. coli* ribosome (EMDB 14850) [19] using ChimeraX [50]. Following this global fit, we performed a localized refinement using a simulated density map of the reference *E. coli* ribosome tunnel, generated in ChimeraX at 6 Å resolution. This step optimized the alignment specifically within the exit tunnel region. Alternatively, ribosome structures can be efficiently aligned using the RADtool VMD plugin (https://radtool.rc.northeastern.edu/).

To validate this density-based alignment, we calculated the root-mean-square deviations (RMSDs) of the conserved PTC residues (as defined in [51]) between the reference *E. coli* structure and each aligned ribosome (Supplementary Fig. S1A). Once aligned, tunnel-lining residues were identified in each structure using VMD by selecting atoms located within the volume of the reference *E. coli* tunnel density. All selections were visually validated in ChimeraX; in cases of significant structural divergence, the selection was manually extended based on visual inspection in VMD.

### Modelling of the missing residues of the exit tunnels

The majority of the generated ribosome tunnel structures (49/64) were complete; however, in a few cases, modelling of missing regions was required (Supplementary Table S5). For ribosomes from *Deinococcus radiodurans, Flavobacterium johnsoniae, Heterocephalus glaber*, and *Staphylococcus aureus*, a single nucleotide (U2585, U2583, U4531, and U2612, respectively) was removed as it overlapped with the C-terminal residue of the NC. This minor modification did not affect the functional shape of the tunnel. The ribosome structures from *F. johnsoniae, Encephalitozoon cuniculi, Giardia lamblia, Neurospora crassa, Plasmodium falciparum, Spraguea lophii, Toxoplasma gondii, Vairimorpha necatrix, Porphyromonas gingivalis, Streptomyces fradiae, Vibrio natrigens*, and *H. marismortui* contained gaps in various protein or rRNA regions within the tunnel (Supplementary Table S5), which were modelled using AlphaFold 2 [52] and AlphaFold 3 [53]. Additionally, certain structures required the removal of obstructing factors prior to simulation: the dormancy factor MDF2 was removed from the *V. necatrix* ribosome, the protein Dap1 was removed from the *Xenopus laevis* tunnel, and an NC density was removed from the *Rattus norvegicus* structure.

### Building starting models for the MD simulations

For each ribosome exit tunnel model, we generated five RNC starting structures with poly-alanine NCs of distinct lengths: 10, 20, 30, 40, and 60 amino acids. The N-terminal residue was modelled based on the ribosome’s biological origin: methionine for eukaryotic and archaeal ribosomes and formylmethionine for bacterial, consistent with the translation initiation in each organism. A proline residue was placed at the C-terminus, following the initial NC model from the *E. coli* ribosome structure (PDB ID: 7zp8) [19], and was held fixed during the simulation. Certain ribosome structures required additional modifications to ensure compatibility with the Amber force fields used in the SBM simulations. Specifically, if parameters for a post-transcriptional modification were unavailable in Amber, the residue was mutated back to its unmodified state.

### MD simulations of the RNCs

Each RNC was simulated three times (replicas) for 5 × 10^7^ time steps in GROMACS 2021 [48] using an all-atom SBM generated via SMOG2 (template: SBM_AA-amber-bonds) [45–47]. The starting NC conformation for the first replica was derived from the reference NC (PDB ID: 7zp8), with minor manual adjustments in Coot [54] to resolve steric clashes as needed. To enhance sampling efficiency, starting NC conformations for the two subsequent replicas were selected from the initial trajectory, based on backbone RMSD; we specifically chose frames that were structurally most distinct from each other and from the initial starting structure. This approach ensured that each simulation was initiated from a very different point in conformational space, facilitating robust sampling of the exit tunnel volume. Throughout the simulations, the ribosomal atoms were held fixed, and the interactions between the NC and the ribosome were treated as purely steric.

### Trajectory analysis

In our approach, we used the NC dynamics to define the functional shape of the ribosomal tunnel. Each NC trajectory was aligned to the starting conformation using the C-terminal proline residue as a reference, enabling direct structural comparison across all systems. Subsequently, each replica trajectory was converted into a volumetric occupancy map using a defined 3D grid (voxel size = 1Å), from which an average occupancy map was calculated. This average map effectively serves as a “plaster cast” of the ribosomal tunnel, delineating all the regions accessible for the NC inside the ribosome. We divided this volume into “edge” voxels that follow the contours of the tunnel walls, and “interior” voxels, based on their location within the map. The edge voxels were used to identify the ribosomal residues lining the tunnel, which were then classified into polar, hydrophobic, positively charged, negatively charged, special residues (Gly, Pro, and Cys) and RNA. Additionally, from each trajectory, we determined the path of maximum occupancy, defined by the voxels within the maximum occupancy for each residue (Cɑ atoms). We then applied cubic B-spline interpolation to this pathway to refine the main path inside the exit tunnel and calculated the average pathway for each ribosome across the three replicas.

### Properties of cytoplasmic ribosome exit tunnels

To systematically compare the structural and physicochemical features of ribosome exit tunnels across bacteria, archaea and eukaryotes, we defined a single reference tunnel path that captures the common exit pathway shared by these ribosomes. We selected the tunnel centerline from the microsporidian *E. cuniculi*, as its geometry is relatively linear and free of major twists or bends within and beyond the vestibule (Supplementary Fig. S4). This linearity minimizes distortions when calculating the perpendicular cross-sectional area along the tunnel, enabling more accurate dimensional comparisons. In addition to cross-sectional area, which serves as our proxy for the tunnel geometry, we examined tunnel asphericity and how key molecular properties change along this reference path. Specifically, we mapped the spatial distribution of polar, hydrophobic, positively and negatively charged residues, and chemically distinct amino acids (Gly, Cys, and Pro). We also calculated the local protein-to-RNA composition ratio, providing insight into the changing molecular environment encountered by the NC during translation.

### Statistical analysis

All structural analyses and MD simulations were performed on representative cytosolic ribosomal structures from across the three domains of life (20 Bacteria, 6 Archaea, and 38 Eukaryota). Each ribosomal tunnel was simulated across five NC lengths (10, 20, 30, 40, and 60 aa) using three independent simulation replicates per setup (*n* = 3), each initiated from a distinct starting NC conformation. Final organism-level results, such as tunnel length, number of specific ribosome contacts, and cross-sectional area, are presented as the arithmetic mean of these replicates.

Measures of domain-specific properties are presented as the domain-wide arithmetic mean, with variation reported as the standard deviation (±SD) across species within each respective domain. In terms of data visualization, tunnel length is plotted as box plots displaying the median and quartiles of the organismal means for each domain. Both the cross-sectional area profiles and the physicochemical profiles are presented as continuous arithmetic means, with colour-shaded boundaries indicating the spread (±SD) across the simulated species. As this study focuses on the descriptive mapping of structural and evolutionary divergence, formal comparative null-hypothesis testing (such as calculating *P*-values) was not performed.

## Results

### Comprehensive identification of ribosome structures across the tree of life

Recent advances in cryo-EM [55, 56] have led to a significant increase in high-resolution ribosome structures, enabling detailed comparative analysis. To systematically explore the structural diversity of ribosomal exit tunnels across the tree of life, we conducted a comprehensive search of the PDB [43] (see the ‘Materials and methods’ section). To ensure the absolute completeness of our dataset, we cross-referenced our findings with the Ribosome Analysis Database (https://radtool.rc.northeastern.edu/). This search identified 78 unique high-resolution structures: 21 bacterial, 51 eukaryotic (including 11 mitochondrial and two chloroplast ribosomes), and 6 archaeal (Supplementary Tables S1–S4). For the present study, we focused on the 65 cytoplasmic ribosomes, as organellar ribosomes, particularly mitochondrial ones, exhibit highly divergent tunnel architectures and were therefore excluded from the analysis. The ribosome from *C. acnes* was also omitted due to the presence of a bound antibiotic in the exit tunnel, which would interfere with structural analysis, leaving us with a final set of 64 ribosomes.

### Tunnel alignment and PTC structural conservation across species

**Figure 1.**
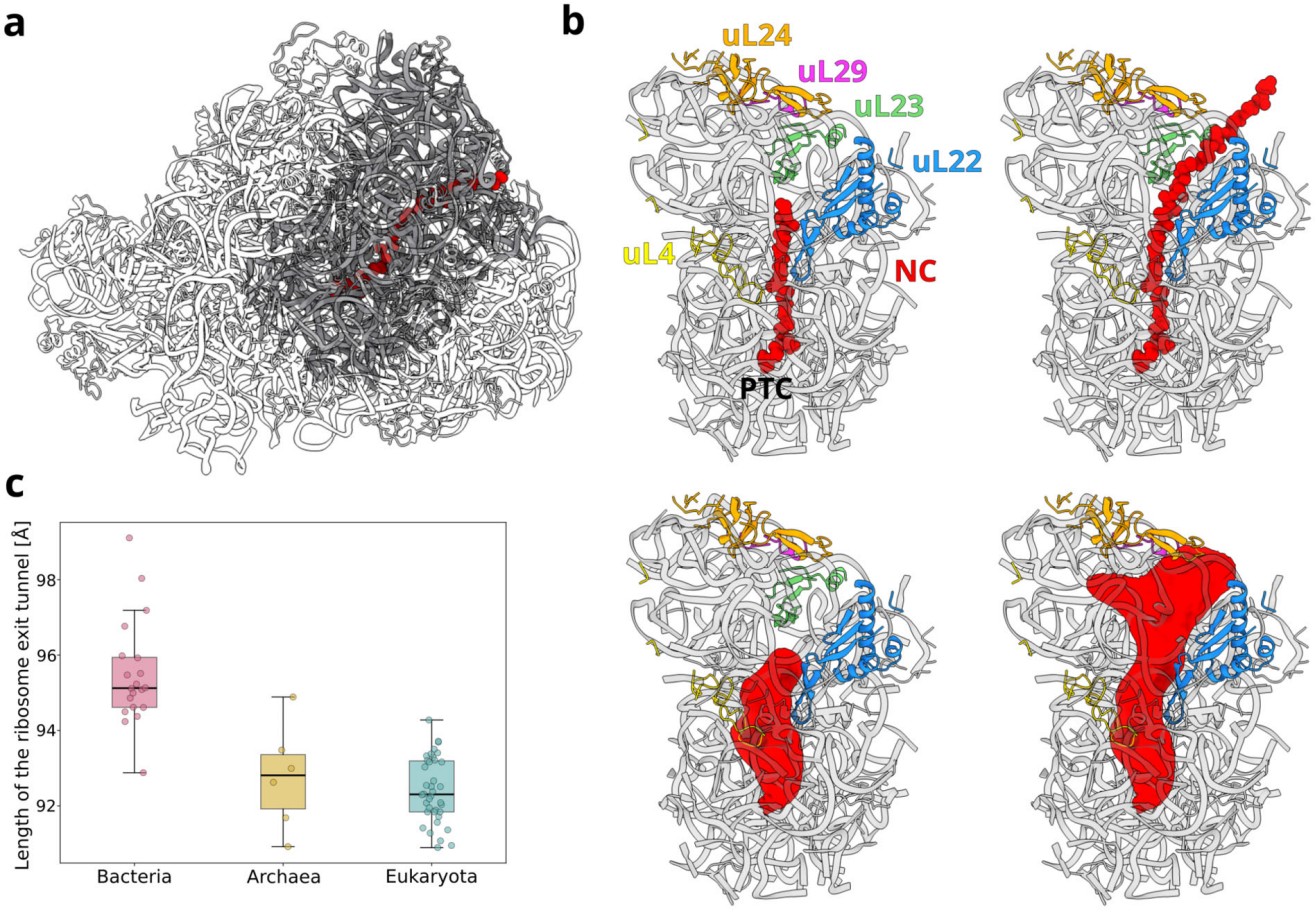
Structural definition of the ribosomal exit tunnel. (**A**) The *E. coli* 50S ribosome structure (PDB ID: 7zp8) [19], highlighting the region selected as the reference exit tunnel with a model of the NC in red. (**B**) Visualization of the functional tunnel definition. Top: The representative snapshots of an NC (red atoms) within the exit tunnel at two different stages of biosynthesis. Bottom: The corresponding 3D occupancy maps (red volume) derived from MD trajectories, capturing the functional volume accessible to the NC. Ribosomal proteins are coloured: uL4 (yellow), uL22 (blue), uL23 (green), uL24 (orange), and uL29 (violet); the position of the PTC is marked. (**C**) Comparison of ribosomal exit tunnel lengths across the three main domains of life.

To enable direct structural comparisons of ribosomal exit tunnels, we aligned all selected ribosome structures to the *E. coli* reference tunnel (PDB ID: 7zp8) [19] and extracted the corresponding tunnel regions (Fig. 1A; see the ‘Materials and methods’ section). Analysis of the PTC, which is the most conserved region of the tunnel, confirmed the high quality of this structural alignment, with average backbone RMSDs of <2 Å relative to *E. coli* for nearly all structures (Supplementary Fig. S1A and C). Despite this high structural similarity, which lies well within the resolution limits of the cryo-EM maps, clustering analysis broadly recapitulates established phylogenetic relationships (Supplementary Fig. S1B). A notable exception was the archaeal ribosome from *Thermococcus kodakarensis*, which displayed a higher RMSD of 3.24 Å (Supplementary Fig. S1C), primarily due to an unusual 23S rRNA conformation (nucleotides 2170–2173; see below).

### A functional definition reveals conserved tunnel lengths across domains

To define the ribosomal exit tunnel in a functionally relevant manner, we adopted an NC-centric approach that captures the accessible volume and boundaries of the tunnel based on NC dynamics, moving beyond the static geometric criteria used in previous studies [39, 41] (Fig. 1B and Supplementary Fig. S2). We hypothesized that tunnel accessibility is dynamic: in the early stages of biosynthesis, distal regions of the tunnel are unreachable to the NC, whereas longer chains become entropically restricted from re-entering deeper segments. To capture this progressive evolution of accessible space, we generated five RNCs with chains of increasing lengths (10, 20, 30, 40, and 60 residues; Supplementary Fig. S2).

By subjecting these complexes to all-atom SBM MD simulations, we calculated average NC occupancy maps that define the functional shape of the tunnel—effectively providing a “plaster cast” of the NC-accessible space (Fig. 1B and Supplementary Fig. S2). This tunnel definition is obtained for a rigid ribosome and is based solely on steric NC-ribosome interactions, thereby providing a baseline for further, more detailed tunnel analysis. Based on these maps, we defined the main tunnel path and used it to quantify changes in tunnel dimensions (length, cross-sectional area, and asphericity) and physicochemical properties along the axis.

Establishing a consistent tunnel boundary across species presents a specific challenge: while the entrance is clearly marked by the 3′ end of the P-site transfer RNA, the exit opens gradually into the wider vestibule. We selected the plane of the uL24 loop, which prominently overhangs the vestibule in bacteria, as this universal marker (Supplementary Fig. S3). Using this definition, we measured the length of each tunnel along its main path (Fig. 1C). In line with previous findings [39], bacterial exit tunnels are slightly longer (mean length: 95.5 ± 1.4 Å) than their eukaryotic (92.4 ± 0.8 Å) and archaeal (92.8 ± 1.3 Å) counterparts. This difference arises primarily from the extended uL24 loop in bacteria, which redirects the NC path and causes it to bend within the vestibule (Supplementary Fig. S4A), whereas the core tunnel path remains highly conserved across all domains (Supplementary Fig. S4B).

### Structural features of bacterial ribosome tunnels

**Figure 2.**
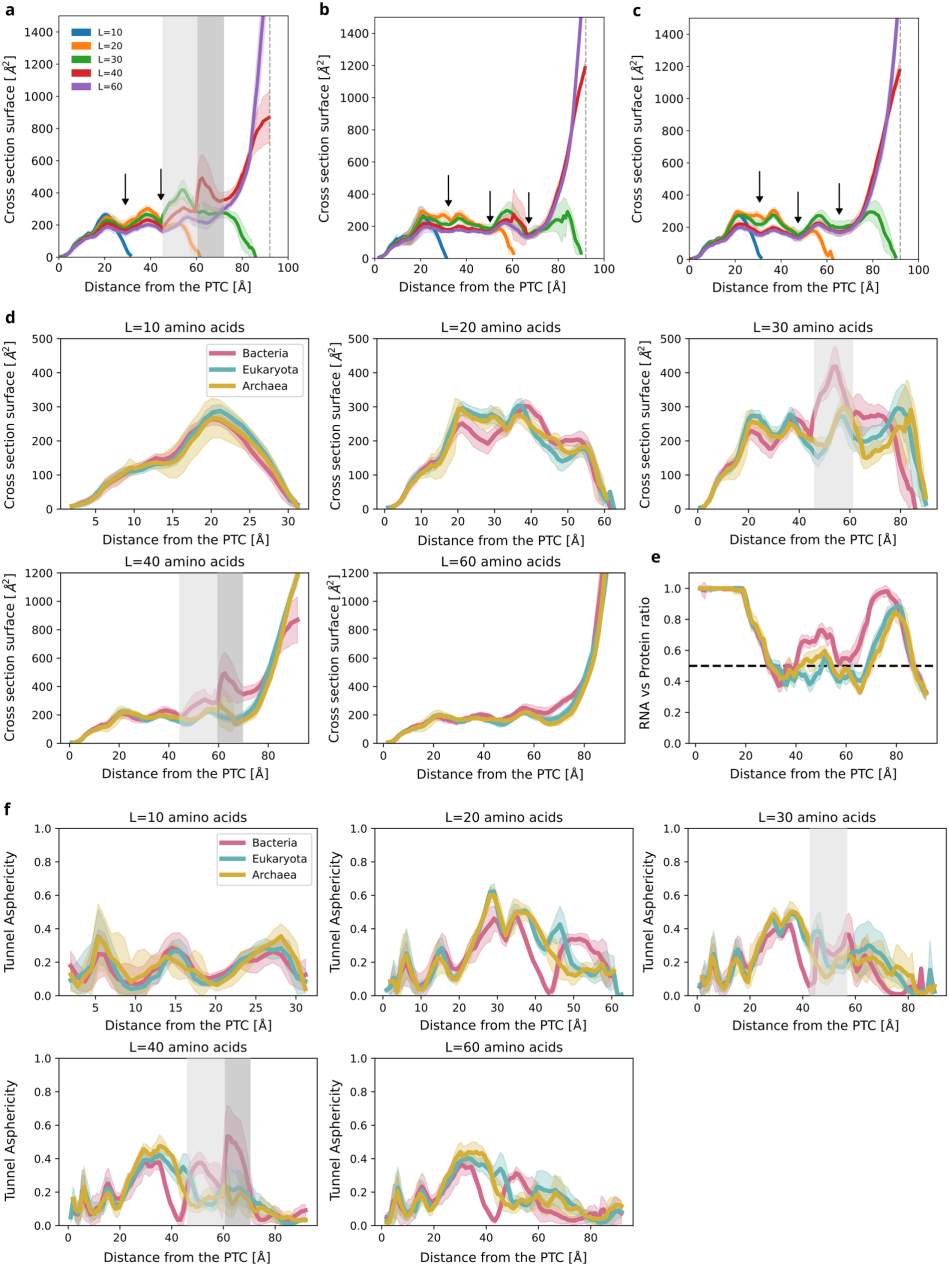
Evolutionary divergence of ribosomal exit tunnel geometry. (**A–C**) Functional cross-sectional area profiles for (**A**) bacteria, (**B**) archaea, and (**C**) eukaryotes, derived from MD simulations of NCs with increasing length (L = 10–60 amino acids). Colour-shaded regions represent the spread across species, whereas the light and dark grey vertical regions represent the first and second lateral branches, respectively. The constriction sites discussed in the text are indicated by black arrows. (**D**) Cross-domain comparison of tunnel profiles for each NC length, highlighting conserved constriction sites and lineage-specific differences. (**E**) Tunnel wall composition mapped along the path, plotted as the ratio of rRNA to ribosomal protein. (**F**) Asphericity profiles quantifying the deviation from circular geometry (0 = circular; 1 = highly elongated), revealing domain-specific topological irregularities.

We characterized the tunnel landscape for all 20 bacterial ribosome tunnels spanning seven phyla (Pseudomonadota, Spirochaetota, Bacillota, Actinomycetota, Deinococcota, Bacteroidota, and Mycoplasmatota), providing broad phylogenetic coverage (Supplementary Fig. S5). Despite this diversity, we observe that bacterial tunnels share a highly conserved core architecture with distinct, transiently accessible features (Fig. 2A and Supplementary Fig. S6). From the perspective of the NC, the first major structural hurdle is a well-characterized constriction site located ∼30 Å from the PTC (Fig. 2A and D). Formed by the extended loops of uL4 and uL22, this narrowing is universal in bacteria and is consistent with previous geometric analyses [39]. However, we identified significant local divergence in this region: ribosomes from *T. thermophilus, M. smegmatis*, and *E. faecalis* display a markedly tighter constriction at ∼27 Å from the PTC (Supplementary Figs S5 and S6A). This distinct narrowing arises from alternative conformations of residues corresponding to *E. coli* Arg61 (uL4) and U790 (23S rRNA), which sterically occlude this exit tunnel region (Supplementary Fig. S7A). Whether this is a species-specific feature or reflects local conformational dynamics remains unknown, but it highlights how even subtle side-chain rearrangements can drastically modulate the effective geometry experienced by the NC. A second constriction is located ∼45 Å from the PTC (Fig. 2A), defined by uL22 residues and surrounding rRNA (Supplementary Fig. S7B). While this aligns positionally with the second constriction site observed in eukaryotes and archaea (∼50 Å from the PTC; see below), it remains significantly wider in bacteria.

**Figure 3.**
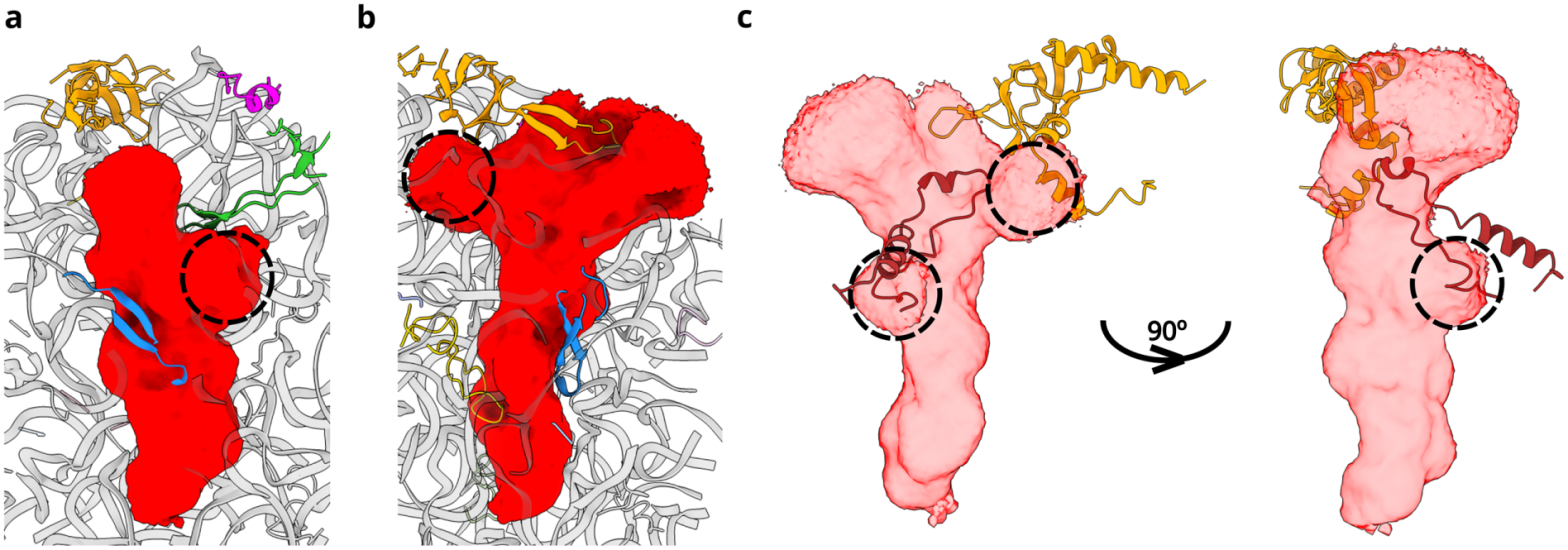
Characterization of bacterial lateral branches and their evolutionary occlusion. (**A**) Location of the first bacteria-specific lateral branch (black dotted circle) relative to the functional tunnel volume (red density) at an NC length of L = 30. (**B**) Location of the second lateral branch (black dotted circle) at L = 40. Ribosomal proteins are coloured: uL4 (yellow), uL22 (blue), uL23 (green), uL24 (orange), and uL29 (violet). (**C**) Superposition of eukaryotic *(H. sapiens*) proteins eL39 (brown) and uL24 (orange) onto the *E. coli* functional tunnel volume (L = 40, red density), demonstrating the steric obstruction of the bacterial lateral branch loci by eukaryotic-specific elements.

Beyond these conserved constrictions, our functional mapping revealed that tunnel accessibility is strictly modulated by NC length, uncovering two previously underappreciated lateral branches (Fig. 2A and D). First, we identified a proximal lateral branch 45–60 Å from the PTC, located beneath the extended loop of the uL23 protein (Figs 2A and 3A). Although this branch is “closed” (it does not breach the ribosome surface), it acts as a significant volumetric expansion. Crucially, this region is particularly accessible to NCs of 30 residues but becomes less available as the chain lengthens, though it remains partially accessible at 40 residues in some species (Supplementary Fig. S8). Access to this lateral branch appears to be gated by the side chains neighbouring rRNA and uL23 residues; for example, in *Mycobacterium tuberculosis*, the protrusion of Arg66, Arg68, and Tyr73 restricts the NC entry (Supplementary Fig. S8B), suggesting a species-specific modulation of accessible space. Second, we observed a larger, distal lateral branch 60–70 Å from the PTC, opening between ribosomal helices H7 and H24 (Figs 2A and 3B). This region becomes accessible at an NC length of 40 residues and is a conserved feature across all examined bacteria, with the notable exceptions of *Listeria monocytogenes, P. gingivalis*, and *S. fradiae*, where a tighter packing of helices H7 and H24 occludes the opening (Supplementary Fig. S9). Particularly noteworthy is the *P. gingivalis* ribosome [57], which features an extended uL23 loop within the tunnel that likely contributes to the occlusion of this lateral branch.

To quantify the resulting deviation from a cylindrical geometry, we calculated the 2D asphericity of the tunnel cross-sections (Fig. 2F). Asphericity values near 0 indicate circular shapes, while values near 1 indicate highly elongated or irregular cross-sections. Across bacterial tunnels, asphericity values ranged from 0 to 0.55, confirming that a simple cylindrical tube model is not an accurate representation of the ribosomal exit tunnel geometry. The regions of highest irregularity coincide with the first constriction site and the two newly identified lateral branches. Conversely, the most circular regions are found close to the PTC, within the vestibule, and at ∼42 Å from the PTC (Fig. 2F), formed by uL22 and uL4 loops as well as rRNA.

Taken together, these results demonstrate that bacterial ribosome tunnels possess both highly conserved features, such as constriction sites, and distinct bacteria-specific lateral branches that are accessible only at specific stages of protein synthesis. These features influence the progression of NCs differently depending on chain length and position within the tunnel. The size, shape, and accessibility of these regions are likely influenced not only by sequence variation in ribosomal proteins and rRNA but also by post-translational and post-transcriptional modifications, as well as local dynamics. These findings reveal a nuanced and temporally regulated landscape that modulates the movement of nascent protein chains through the bacterial ribosome tunnel.

### Archaeal ribosome tunnels exhibit distinct structural adaptations

We next analysed the exit tunnels of six archaeal ribosomes representing two major phyla: Euryarchaeota and Thermoproteota (Supplementary Figs S10 and S11). As in bacteria, the functional volume of the tunnel changes as the NC lengthens (Fig. 2B and Supplementary Fig. S11), although the transitions appear less pronounced in archaea. The unique structural features of archaeal tunnels become especially apparent when compared to their bacterial counterparts (Fig. 2D).

Near the PTC, archaeal tunnels closely resemble bacterial ones, reflecting strong evolutionary conservation at both the sequence and structural levels (Fig. 2D). However, the first constriction site (∼30 Å from the PTC) is notably wider in archaea (Fig. 2D). This difference likely arises from the absence of a conserved arginine (equivalent to *E. coli* Arg61) that protrudes into the tunnel in bacteria but is missing in archaea (Supplementary Fig. S12A). Moving further downstream, the tunnel architecture diverges significantly due to the incorporation of the archaeal/eukaryotic-specific protein eL39 and the presence of a distinct second loop in uL4 (absent in bacteria). These elements effectively remodel the tunnel wall, shifting the second constriction site ∼5 Å downstream (to ∼50 Å from the PTC) compared to bacteria (Fig. 2B and Supplementary Fig. S13C).

Crucially, the structural addition of eL39 acts as an evolutionary “plug” that seals both lateral cavities observed in bacteria (Fig. 3C). The region corresponding to the bacterial first lateral branch is mainly occluded by eL39 (Fig. 3C and Supplementary Fig. S12B), which packs against uL22 and rRNA, creating a narrower constriction in archaea than in bacteria at this depth (Fig. 2D). Similarly, the second lateral branch (60–70 Å) is effectively sealed by eL39 with the help of conserved archaeal/eukaryotic-specific N-terminal extension to the uL24 (Fig. 3C and Supplementary Fig. S14), resulting in a third, highly pronounced constriction (Fig. 2B and Supplementary Fig. S12C). Notably, the positioning of eL39 in this region directly intersects with the H6/H7 NC pathway recently characterized in other systems [19, 42], suggesting that this specific pathway may be structurally occluded in the archaeal and eukaryotic ribosomes.

Despite this general trend toward a “sealed” tunnel, we observe two archaeal species that deviate from the general tunnel architecture. In *T. kodakarensis*, an unusual 23S rRNA conformation (nucleotides 2170–2173) blocks a significant portion of the tunnel near the PTC, obstructing the NC’s path (Supplementary Fig. S13A). This unique feature is also present in other available structures of this ribosome (PDB IDs: 6skf and 6skg) [58] but is distinct from the typical archaeal geometry near the PTC, as visible in our RMSD analysis (Supplementary Fig. S1A). Conversely, *Sulfolobus acidocaldarius* retains a lateral branch at a position similar to the bacterial ribosome (L = 40; Supplementary Fig. S10), formed by a different conformation of rRNA and eL39 residues (Supplementary Fig. S13B). Given the limited number of high-resolution archaeal ribosome structures (six species from two phyla), it remains an open question whether these features represent broad lineage-specific diversity or species-specific adaptations.

Finally, the tunnel asphericity in archaea also changes along the tunnel axis (Fig. 2F). The region around the PTC is very similar to the bacterial one, highlighting the structural and sequence conservation of this core region. The main differences correspond to the regions of bacterial-specific cavities, which are more spherical in archaea (at NC lengths of 30 and 40 amino acids). Notably, the region near the second constriction site exhibits significant differences in asphericity, despite less pronounced variations in cross-sectional area (Fig. 2D and E). This difference arises from the insertion of the second uL4 loop, which alters the shape—but not necessarily the overall cross-sectional area (Supplementary Fig. S13C).

### Structural features of eukaryotic ribosome tunnels and the divergence of Microsporidia

Eukaryotic ribosomes comprise the largest group in our analysis, including 38 unique structures from 12 phyla (Ascomycota, Amoebozoa, Nematoda, Arthropoda, Chordata, Microsporidia, Euglenozoa, Fornicata, Streptophyta, Apicomplexa, Ciliophora, and Parabasalia). While the majority of these structures share a highly conserved architecture, our initial analysis revealed that ribosome tunnels from Microsporidia (*E. cuniculi, P. locustae, S. lophii*, and *V. necatrix*) exhibit distinct structural features (Supplementary Figs S15 and S16), consistent with recent observations [41]. Consequently, we analysed these parasitic species separately from the canonical eukaryotic consensus.

Focusing first on the canonical eukaryotic ribosomes, we generated an averaged tunnel cross-sectional area profile, which showed the least variation across the three domains of life (Fig. 2C and Supplementary Fig. S17). Notably, the overall architecture of the eukaryotic tunnel closely mirrors that of archaea, preserving three constriction sites consistently located at ∼30 Å, ∼50 Å, and ∼67 Å from the PTC (Fig. 2C). These narrow regions are formed by the same structural elements found in archaea, most notably the eL39 protein, which occludes the regions corresponding to the two bacterial lateral branches (Fig. 3 and Supplementary Fig. S18) as well as N-terminal extensions of the uL24 that occlude the second lateral branch (Fig. 3 and Supplementary Fig. S14). This high degree of structural similarity between eukaryotic and archaeal exit tunnels, evident in both cross-sectional area and asphericity profiles (Fig. 2F), highlights their common evolutionary roots and confirms that the “sealed” tunnel architecture is a shared evolutionary trait.

In contrast, Microsporidia ribosomes exhibit markedly different tunnel architectures, reflecting the extensive ribosomal reduction typical of their obligate parasitic lifestyle. The extent of ribosome reduction varies across species, ranging from the relatively canonical tunnel of *P. locustae* to the heavily reduced structure in *V. necatrix* (Supplementary Fig. S19). These reductions primarily affect the lower portion of the tunnel, beginning ∼60 Å from the PTC, which becomes significantly more open and solvent-exposed compared to other eukaryotes (Supplementary Figs S15 and S20). This aligns with recent geometric clustering that grouped specific parasitic protists with prokaryotes rather than canonical eukaryotes [41]. While this increased architectural opening may facilitate NC flexibility and earlier folding [19, 20], it likely also exposes the emerging polypeptide to premature interaction with cytosolic factors.

Taken together, our analysis reveals that cytoplasmic ribosome exit tunnels show a high degree of structural conservation within domains but can diverge fundamentally between lineages. This striking structural similarity between archaeal and eukaryotic tunnels, evident in cross-sectional area, asphericity profiles, and the specific “sealing” role of eL39 and N-terminal extensions to uL24, underscores their shared evolutionary history. In contrast, bacterial tunnels exhibit greater structural diversity, particularly in the variable opening of the two lineage-specific lateral branches, contributing to a broader architectural plasticity. This suggests an evolutionary trajectory in which the tunnel began as a variable, multibranched landscape in bacteria but was refined into a more stringent, sealed conduit in the archaeal-eukaryotic lineage.

### The physicochemical landscape of the exit tunnel

Beyond geometric constraints, our computational approach also enabled us to explore the physicochemical environment experienced by the NC as it traverses the ribosomal exit tunnel. We first mapped the rRNA-to-protein ratio along the tunnel path (L = 60 NC simulations; Fig. 2E). While the tunnel is predominantly lined by rRNA, the central region, between the first and third constriction sites, shows a notable increase in protein content, particularly in archaea and eukaryotes. This enrichment arises from the presence of extended loops of uL4, uL22, and uL23 (in bacteria), as well as the eukaryotic- and archaeal-specific second uL4 loop and eL39 protein. Consequently, the tunnel wall in archaea and eukaryotes presents a significantly more protein-rich surface than its bacterial counterpart.

**Figure 4.**
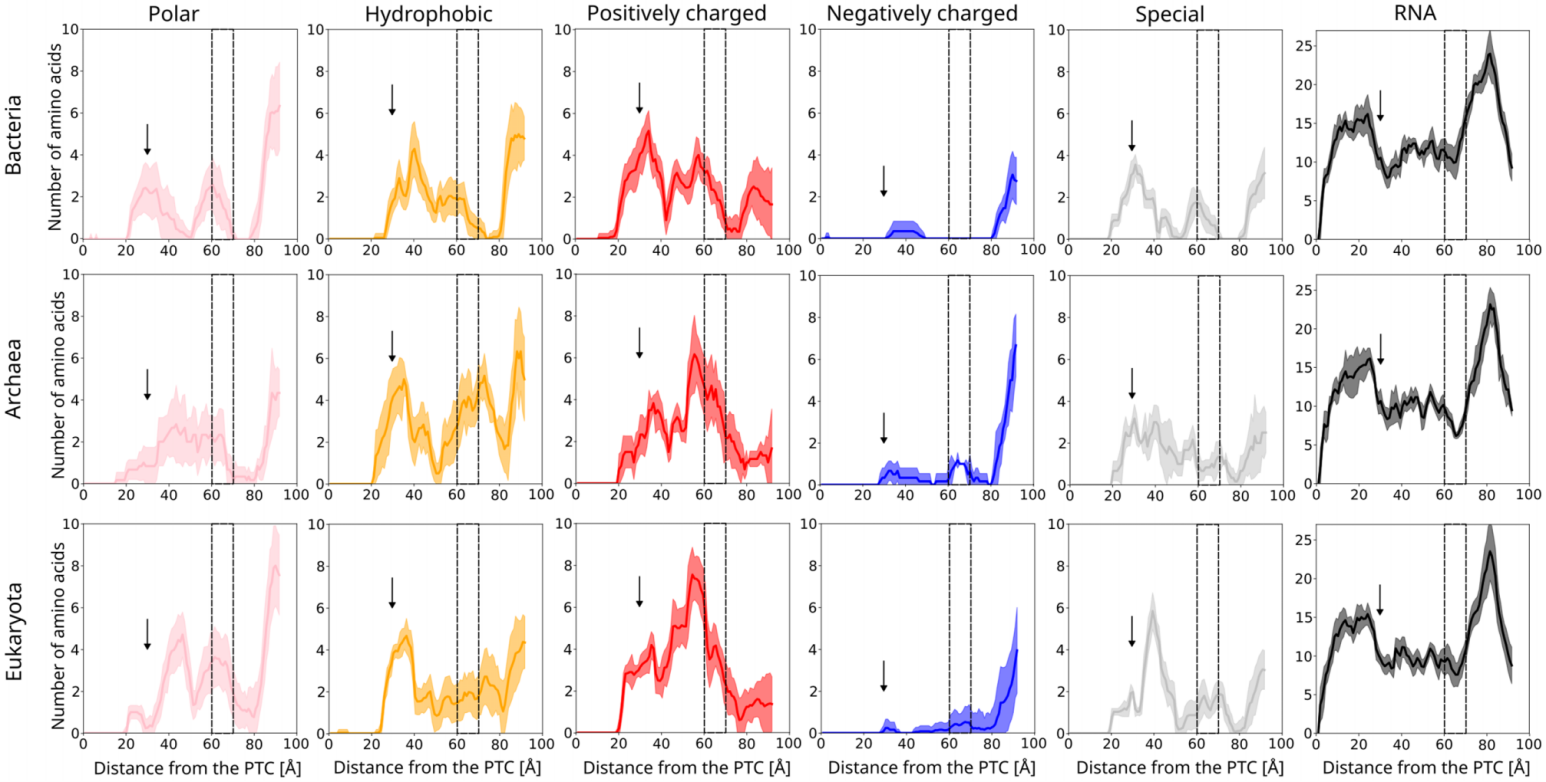
Divergent physicochemical landscape of the ribosomal exit tunnel. Comparison of the chemical environment lining the functional tunnel path across bacteria, archaea, and eukaryotes. The profiles display the contact frequency of rRNA nucleotides and amino acid residues as a function of distance from the PTC. Amino acids are grouped into five physicochemical categories: polar, hydrophobic, positively charged, negatively charged, and special (Gly, Pro, Cys). The position of the first constriction site is highlighted by arrows, and a black dashed rectangle highlights the region 60–70 Å from the PTC (the eL39 binding site). Notably, the archaeal tunnel is more hydrophobic and less polar than the eukaryotic tunnel in the eL39 binding region, while the bacterial tunnel displays a distinct enrichment of polar and positively charged residues at the first constriction site.

To further dissect the tunnel composition, we calculated the number of rRNA nucleotides and specific amino acids [polar, hydrophobic, positively charged, negatively charged, and special (Gly, Pro, and Cys)] lining the main tunnel path (Fig. 4). Strikingly, the rRNA profiles are very similar across all three domains of life. This indicates that the RNA provides a universally conserved scaffold, while the physicochemical differences of the tunnel are driven almost exclusively by protein composition.

Negatively charged residues are largely excluded from the tunnel interior, likely due to electrostatic repulsion from the dense, negatively charged rRNA backbone, and appear predominantly near the tunnel exit. Within the tunnel, the dominant residue types are polar, hydrophobic and positively charged (Fig. 4). Consistent with their structural homology, archaeal and eukaryotic tunnels again display highly similar profiles, with the main difference observed in the region 60–70 Å from the PTC (the eL39 binding site). In the archaeal tunnel, this region is more hydrophobic (3.77 ± 1.19 versus 1.66 ± 0.86 residues; see region highlighted in Fig. 4) and less polar than in eukaryotes (1.52 ± 0.93 versus 3.00 ± 1.24 residues). In contrast, bacterial tunnels, which lack eL39, are generally less hydrophobic (1.19 ± 0.66 residues) and less positively charged in this region (2.12 ± 0.71 versus 3.76 ± 1.39 and 3.52 ± 0.94 residues in archaea and eukaryotes, respectively). An additional difference is observed at the first constriction site, where the bacterial tunnel shows an enrichment of polar residues (2.35 ± 1.19 versus 0.83 ± 0.90 and 0.23 ± 0.42 in archaea and eukaryotes, respectively; see arrows in Fig. 4) and positively charged residues (4.05 ± 0.86 versus 2.33 ± 0.92 and 3.18 ± 0.51 in archaea and eukaryotes, respectively), a feature consistent with the structural divergence noted earlier in this region.

## Discussion

In this study, we developed and applied an NC-centric computational framework to define and analyse the cytoplasmic ribosome exit tunnel across the tree of life. Unlike previous geometry-based approaches [39, 41, 59], our method establishes a functional definition of the tunnel, based on the steric and entropic accessibility of the nascent polypeptide itself. Moreover, by studying the ribosomal tunnel at different time points during protein synthesis, we observe how ribosome features can be temporally gated by NC length. This shift enables a natural delineation of tunnel boundaries and reveals transiently accessible features, most notably the bacteria-specific lateral branches that have not been previously characterized.

We demonstrate that the tunnel has a highly complex and irregular geometry, characterized by a nonlinear axis and variable, noncircular cross-sections (high asphericity; Fig. 2). This topological complexity precluded the use of simple radial measurements to accurately characterize tunnel dimensions. Instead, we propose functional cross-sectional area as the biologically relevant metric, which provides a more accurate proxy for the space accessible to the NC. Consequently, our area-derived values are generally larger than those inferred from the commonly cited 10–20 Å radius range, as they account for irregular cavities, pockets and lateral branches along the path.

Our functional mapping provides a mechanistic explanation for the geometric divergence observed between domains. While previous studies described the eukaryotic tunnel as “narrower” due to a second constriction [39], our analysis reveals that this narrowing results also from a specific evolutionary strategy: the “plugging” of ancestral side cavities. We show that the lateral branches accessible in bacteria are effectively sealed in archaea and eukaryotes by the incorporation of eL39 and an N-terminus extension of the protein uL24 (Fig. 3 and Supplementary Fig S14). This suggests an evolutionary trajectory in which the tunnel transitioned from a multibranched, porous landscape in bacteria to a strictly confined conduit in higher organisms. This architectural “tightening” aligns with the hypothesis that the eukaryotic tunnel acts as a selective filter to trap specific small proteins [40]. Furthermore, our observation that parasitic Microsporidia “re-open” these distal regions supports the recent finding that tunnel geometry is plastic and can revert to a bacterial-like state under specific evolutionary pressures [33]; however, our observation suggests that the nature of the widening of the tunnel is very different from that of bacteria.

The exit tunnel vestibule is a critical environment in which proteins can initiate folding into native or near-native compact structures [10, 11, 13]. The ribosome actively modulates this process, not only through direct enthalpy-driven interactions with the growing NC [60–64], which can destabilize the native state [19, 65], but also through entropic destabilization of the disordered ensemble [66]. By restricting the conformational space available to the unfolded chain, the tunnel lowers the conformational entropy of the disordered state, thereby stabilizing compact folding intermediates and altering potential folding pathways [66–68]. Consequently, the specific shape and dimensions of the exit tunnel directly define this entropic landscape, likely tuning the trajectory of co-translational folding.

While structural variability may arise from different ribosome functional states (e.g. empty versus translating) or variations in experimental methods and conditions, our PTC RMSD analysis (Supplementary Fig. S1) and low variance across cross-sectional profiles (Fig. 2D) suggest these effects are minimal. However, an open question remains regarding how ribosomal dynamics, especially side-chain motion, can modulate its accessibility to the NC in real time. For instance, we have already observed that small changes in side-chain orientation can have a significant impact on the effective geometry experienced by the NC. Additionally, it has been shown that ribosome dynamics (e.g. the uL22 protein) can facilitate allosteric communication between the ribosome surface and its interior [69], pointing to an important functional aspect. Incorporating ribosomal flexibility into future simulations will allow us to capture these effects more directly, particularly as our strategy employs SBMs, which are well-adapted to various MD scenarios and widely used in ribosome simulations [70, 71].

Finally, our current use of a polyalanine NC sequence, with N-terminal methionine (eukaryotes/archaea) or formylmethionine (bacteria), and steric-only interactions serves as a topological baseline. While the shape of the ribosome tunnels reported here defines the maximal steric boundaries, it should be noted that the actual sampling of this space by a nascent peptide may be nonuniform due to conformational entropy and geometric confinement. We focus on the total accessible volume to provide a sequence-independent baseline that defines the absolute physical constraints of the exit tunnel across different species within which any NC must operate. By comparing this reference map against system-specific maps that incorporate side-chain chemistry, electrostatics, and specific stalling sequences, we can begin to dissect the interplay between tunnel architecture and NC identity. Combining this with ribosome dynamics will open a new window to study these systems. This also enables prediction of the effects of post-translational and post-transcriptional modifications, antibiotic binding, or regulatory nascent peptide sequences—a key step toward predictive models of ribosome-NC interactions.

## Supplementary Material

gkag689_Supplemental_File

## Data Availability

All raw and processed data resulting from the MD simulations, including MD trajectories and the 64 species-specific occupancy maps and calculated cross-sectional area profiles, have been deposited in Zenodo and can be accessed at 10.5281/zenodo.18079792. The code for data analysis is available at https://github.com/dydymos/ribotunnel.
