# Supplementary material for: Evolution of the ribosomal exit tunnel through the eyes of the nascent chain
Source: Nucleic Acids Res. 2026 Jul 7;54(13):gkag689. doi: 10.1093/nar/gkag689 (PMC13338714; doi:10.1093/nar/gkag689)
Supplement: gkag689_Supplemental_File [file gkag689_supplemental_file.pdf]

# Evolution of the ribosomal exit tunnel through the eyes of the nascent chain

## Supplementary Tables

| ID | Organism | Phylum | Type of ribosome | PDB ID | Resolution [Å] |
|---|---|---|---|---|---|
| *1* | *A.baumannii* | Pseudomonadota | P-site tRNA | 6v39 | 3.04 |
| *2* | *B.burgdorferi* | Spirochaetota | empty ribosome | 8fn2 | 3.40 |
| *3* | *B.subtilis* | Bacillota | P-site tRNA | 7as8 | 2.90 |
| *4* | *C.acnes* | Actinomycetota | P-site tRNA | 8crx | 2.78 |
| *5* | *D.radiodurans* | Deinococcota | empty ribosome | 5dm6 | 2.90 |
| *6* | *E.coli* | Pseudomonadota | P-site tRNA | 7zp8 | 2.20 |
| *7* | *E.faecalis* | Bacillota | P-site tRNA | 7p7u | 3.10 |
| *8* | *F.johnsoniae* | Bacteroidota | empty ribosome | 7jil | 2.80 |
| *9* | *L.innocua* | Bacillota | P/E-site tRNA | 8uu5 | 3.00 |
| *10* | *L.lactis* | Bacillota | empty ribosome | 5myj | 5.60 |
| *11* | *L.monocytogenes* | Bacillota | empty ribosome | 8a63 | 3.10 |
| *12* | *M.pneumoniae* | Mycoplasmatota | A- P-site tRNAs | 7pal | 4.70 |
| *13* | *M.smegmatis* | Actinomycetota | P-site tRNA | 8fr8 | 2.76 |
| *14* | *M.tuberculosis* | Actinomycetota | P- E-site tRNAs | 7mt7 | 2.71 |
| *15* | *P.aeruginosa* | Pseudomonadota | empty ribosome | 6spd | 3.28 |
| *16* | *P.gingivalis* | Bacteroidota | empty ribosome | 9i5x | 3.00 |
| *17* | *P.urativorans* | Pseudomonadota | P-site tRNA | 8rdv | 2.60 |
| *18* | *S.aureus* | Bacillota | empty ribosome | 6hma | 2.65 |
| *19* | *S.fradiae* | Actinomycetota | empty ribosome | 9qt5 | 3.13 |
| *20* | *T.thermophilus* | Deinococcota | A- P- E-site tRNAs | 8cvl | 2.30 |
| *21* | *V.*natrigens | Pseudomonadota | empty ribosome | 9h91 | 2.70 |

**Supplementary Table S1**. List of bacterial ribosome structures selected from the PDB search.

| ID | Organism | Phylum | Type of ribosome | PDB ID | Resolution [Å] |
|---|---|---|---|---|---|
| *1* | *A.thaliana* | Streptophyta | P- E-site tRNA | 9h3g | 1.82 |
| *2* | *C.albicans* | Ascomycota | empty ribosome | 7pzy | 2.32 |
| *3* | *C.elegans* | Nematoda | A- P-site tRNAs | 9bh5 | 2.63 |
| *4* | *C.porcellus* | Chordata | P/E-site tRNA | 9y4h | 4.70 |
| *5* | *C.sericeus* | Chordata | P- E-site tRNAs | 9y49 | 3.40 |
| *6* | *C.thermophilia* | Ascomycota | P/E-site tRNA | 7old | 3.00 |
| *7* | *D.melanogaster* | Arthropoda | E-site tRNA | 4v6w | 6.00 |
| *8* | *D.rerio* | Chordata | empty ribosome | 7oyb | 2.40 |
| *9* | *E.cuniculi* | Microsporidia | empty ribosome | 7qep | 2.70 |
| *10* | *E.gracilis* | Euglenozoa | A- P- E-site tRNAs | 6zj3 | 3.15 |
| *11* | *E.histolytica* | Amoebozoa | P-site tRNA | 9v21 | 3.00 |
| *12* | *G.gallus* | Chordata | E-site tRNA | 8q7z | 2.50 |
| *13* | *G.lamblia* | Fornicata | empty ribosome | 7pwg | 2.75 |
| *14* | *H.glaber* | Chordata | E-site tRNA | 9y44 | 4.90 |
| *15* | *H.sapiens* | Chordata | A- P-site tRNAs | 8jdk | 2.26 |
| *16* | *K.lactis* | Ascomycota | P/E-site tRNA | 6uz7 | 3.60 |
| *17* | *L.donovani* | Euglenozoa | empty ribosome | 5t2a | 2.90 |
| *18* | *L.major* | Euglenozoa | empty ribosome | 8a3w | 2.89 |
| *19* | *M.musculus* | Chordata | E-site tRNA | 7ls2 | 3.10 |
| *20* | *N.crasa* | Ascomycota | A- P-site tRNAs | 7r81 | 2.70 |
| *21* | *N.tabacum* | Streptophyta | A/P- P/E-site tRNAs | 8b2l | 2.20 |
| *22* | *O.cuniculus* | Chordata | P-site tRNA | 6sgc | 2.80 |
| *23* | *P.falciparum* | Apicomplexa | P-site tRNA | 3jbn | 4.70 |
| *24* | *P.locustae* | Microsporidia | empty ribosome | 6zu5 | 2.90 |
| *25* | *R.norvegicus* | Chordata | A/P- P/E-site tRNAs | 7qgg | 2.86 |
| *26* | *S.cerevisiae* | Ascomycota | A/P- P/E-site tRNAs | 8ccs | 1.97 |
| *27* | *S.lophii* | Microsporidia | empty ribosome | 7qca | 2.79 |
| *28* | *S.lycoperscium* | Streptophyta | A- P-site tRNAs | 7qiz | 2.38 |
| *29* | *S.pombe* | Ascomycota | empty ribosome | 8eug | 2.80 |
| *30* | *S.scrofa* | Chordata | empty ribosome | 3j7p | 3.50 |
| *31* | *T.aestivum* | Streptophyta | P-site tRNA | 4v7e | 5.50 |
| *32* | *T.brucei* | Euglenozoa | empty ribosome | 8ova | 2.47 |
| *33* | *T.cruzi* | Euglenozoa | empty ribosome | 5t5h | 2.54 |
| *34* | *T.gondii* | Apicomplexa | empty ribosome | 5xxb | 3.17 |
| *35* | *T.thermophila* | Ciliophora | empty ribosome | 4v8p | 3.52 |
| *36* | *T.vaginalis* | Parabasalia | empty ribosome | 5xy3 | 3.20 |

| | | | | | |
|---|---|---|---|---|---|
| *37* | *V.necatrix* | Microsporidia | empty ribosome | 6rm3 | 3.40 |
| *38* | *X.laevis* | Chordata | empty ribosome | 7oyc | 2.40 |

**Supplementary Table S2.** List of cytoplasmic eukaryotic ribosome structures selected from the PDB search.

| ID | Organism | Phylum | Type of ribosome | PDB ID | Resolution [Å] |
|---|---|---|---|---|---|
| *1* | *H.marismortui* | Euryarchaeota | empty ribosome | 1s72 | 2.40 |
| *2* | *M.acetivorans* | Euryarchaeota | empty ribosome | 9zng | 2.06 |
| *3* | *P.calidifontis* | Thermoproteota | empty ribosome | 9e71 | 2.36 |
| *4* | *P.furiosus* | Euryarchaeota | P- E-site tRNAs | 4v6u | 6.60 |
| *5* | *S.acidocaldarius* | Thermoproteota | empty ribosome | 8hku | 2.72 |
| *6* | *T.kodakarensis* | Euryarchaeota | empty ribosome | 6th6 | 2.55 |

**Supplementary Table S3.** List of archaeal ribosome structures selected from the PDB search.

| ID | Organism | Phylum | Type of ribosome | PDB ID | Resolution [Å] |
|---|---|---|---|---|---|
| | ***Mitochondrial*** | | | | |
| *1* | *A. thaliana / B. oleracea* | Streptophyta | empty ribosome | 6xyw | 3.86 |
| *2* | *C. reinhardtii* | Chlorophyta | empty ribosome | 7pkt | 3.00 |
| *3* | *H.sapiens* | Chordata | A- P-site tRNAs | 6zm5 | 2.89 |
| *4* | *L. tarentolae / L. major* | Euglenozoa | empty ribosome | 7aih | 3.60 |
| *5* | *N.crassa* | Ascomycota | P-site tRNA | 6ywy | 3.05 |
| *6* | *P.magna* | Chlorophyta | P-site tRNA | 8apn | 3.10 |
| *7* | *S.cerevisiae* | Ascomycota | P-site tRNA | 5mrc | 3.25 |
| *8* | *S.scrofa* | Chordata | P-site tRNA | 6ydp | 3.00 |
| *9* | *T.brucei* | Euglenozoa | empty ribosome | 6hiv | 7.80 |
| *10* | *T.gondii* | Apicomplexa | empty ribosome | 9g6k | 2.89 |
| *11* | *T. thermophila* | Ciliophora | empty ribosome | 6z1p | 3.70 |

| ID | Organism | Phylum | Type of ribosome | PDB ID | Resolution [Å] |
|---|---|---|---|---|---|
| | ***Chloroplast*** | | | | |
| *1* | *C. reinhardtii* | Chlorophyta | empty ribosome | 9tvu | 2.52 |
| *2* | *S.oleracea* | Streptophyta | empty ribosome | 5x8p | 3.40 |

**Supplementary Table S4.** List of organellar ribosome structures identified in the PDB search.

| Organism | PDB ID | Problems with the exit tunnel region |
|---|---|---|
| *A.baumannii* | 6v39 | None |
| *B.burgdorferi* | 8fn2 | None |
| *B.subtilis* | 7as8 | None |
| *C.acnes* | 8crx | Sarecyclin bound in the exit tunnel |
| *D.radiodurans* | 5dm6 | U2585 was removed to make space for the NC |
| *E.coli* | 7zp8 | None |
| *E.faecalis* | 7p7u | None |
| *F.johnsoniae* | 7jil | uL23 and uL24 loops are missing, modelled based on the AF models; U2583 was removed to make space for NC |
| *L.innocua* | 8uu5 | None |
| *L.lactis* | 5myj | None |
| *L.monocytogenes* | 8a63 | None |
| *M.pneumoniae* | 7pal | None |
| *M.smegmatis* | 8fr8 | None |
| *M.tuberculosis* | 7mt7 | None |
| *P.aeruginosa* | 6spd | None |
| *P.gingivalis* | 9i5x | Copied 1466-1484 and 1485-1595 nt from 9it5 and uL24 loop modelled from AF model |
| *P.urativorans* | 8rdv | None |
| *S.aureus* | 6hma | U2612 was removed to make space for the NC |
| *S.fradiae* | 9qt5 | We modelled missing rRNA sequences 2721-2723 and 2279-2281 bases on a similar ribosome from *C.acnes*; uL24 and uL4 loop modelled from AF model. Need to remove T2804 to make space for NC's Pro |
| *T.thermophilus* | 8cvl | None |
| *V.natrigens* | 9h91 | uL24 loop modelled from AF model |

| Organism | PDB ID | Problems with the exit tunnel region |
|---|---|---|
| *A.thaliana* | 9h3g | None |
| *C.albicans* | 7pzy | None |
| *C.elegans* | 9bh5 | None |
| *C.thermophilia* | 7old | None |
| *C.porcellus* | 9y4h | None |
| *C.sericeus* | 9y49 | None |

| | | |
|---|---|---|
| *D.melanogaster* | 4v6w | None |
| *D.rerio* | 7oyb | None |
| *E.cuniculi* | 7qep | missing a short fragment of the rRNA helix (U1134,G1135); model of the full helix we get from AF3, and upon superposition, we model the missing two nucleotides |
| *E.gracilis* | 6zj3 | None |
| *E.histolytica* | 9v21 | None |
| *G.gallus* | 8q7z | None |
| *G.lamblia* | 7pwg | missing a short fragment of the rRNA (U335-C337) and part of ribosomal protein L31e (D75-K79); modelled with AF2/3 models |
| *H.glaber* | 9y44 | had to remove U4531 - overlap with the C-terminal residue of the NC |
| *H.sapiens* | 8jdk | None |
| *K.lactis* | 6uz7 | None |
| *L.donovani* | 5t2a | None |
| *L.major* | 8a3w | None |
| *M.musculus* | 7ls2 | None |
| *N.crasa* | 7r81 | uL29 loop is missing (S40-L45), we modelled it using the AF2 structure |
| *N.tabacum* | 8b2l | None |
| *O.cuniculus* | 6sgc | None |
| *P.falciparum* | 3jbn | eL39 loop is missing (K30-Y36), we modelled it using the AF2 structure |
| *P.locustae* | 6zu5 | None |
| *R.norvegicus* | 7qgg | None |
| *S.cerevisiae* | 8ccs | None |
| *S.lophii* | 7qca | Modelling of the missing rRNA region (U417-A421) |
| *S.lycoperscium* | 7qiz | None |
| *S.pombe* | 8eug | None |
| *S.scrofa* | 3j7p | None |
| *T.aestivum* | 4v7e | None |
| *T.brucei* | 8ova | None |
| *T.cruzi* | 5t5h | None |
| *T.gondii* | 5xxb | uL4 loop is missing G83, we modelled it using the AF2 structure |
| *T.thermophila* | 4v8p | None |
| *T.vaginalis* | 5xy3 | None |
| *V.necatrix* | 6rm3 | Missing short fragments of rRNA: U258-U260, U1141-U1142 and A1171-U1172 were modelled with AF3. We were not able to model A65-U69, but it is further from the tunnel, so it should not affect the exit tunnel path |
| *X.laevis* | 7oyc | None |

| Organism | PDB ID | Problems with the exit tunnel region |
|---|---|---|
| *H.marismortui* | 1s72 | eL39 has missing region (E33–Q35), we modelled it using the AF2 structure |
| *M.acetivorans* | 9zng | None |
| *P.calidifontis* | 9e71 | None |
| *P.furiosus* | 4v6u | None |
| *S.acidocaldarius* | 8hku | None |
| *T.kodakarensis* | 6th6 | None |

**Supplementary Table S5.** List of structural modifications applied to the ribosome exit tunnel models.

# Supplementary Figures

A)

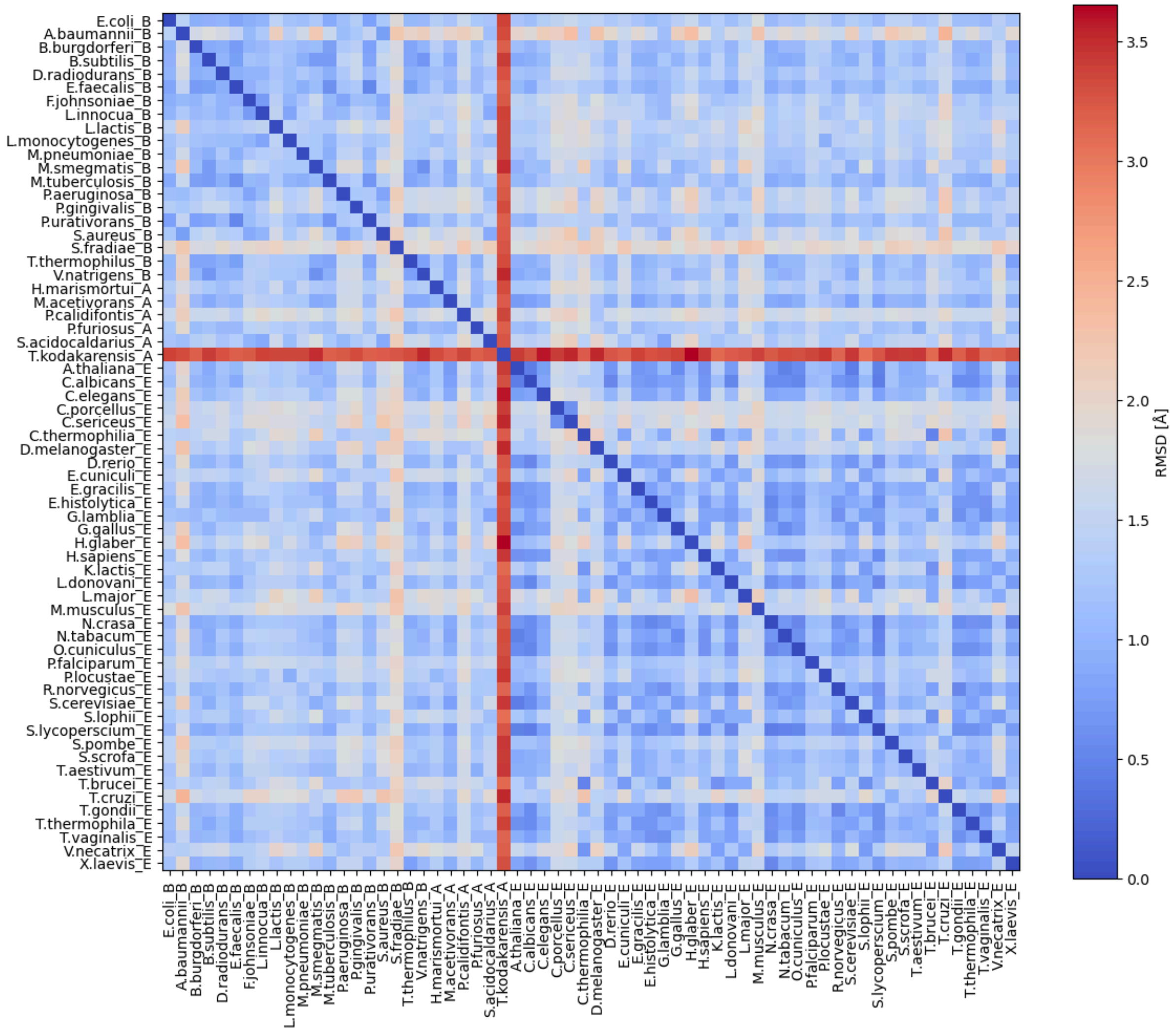

B)

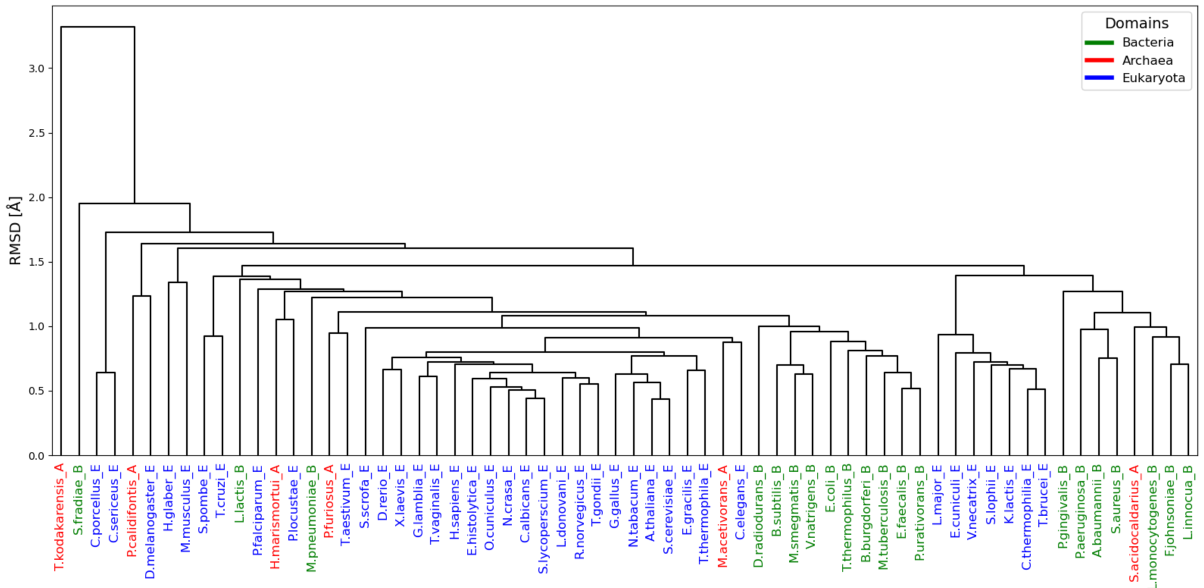


C)

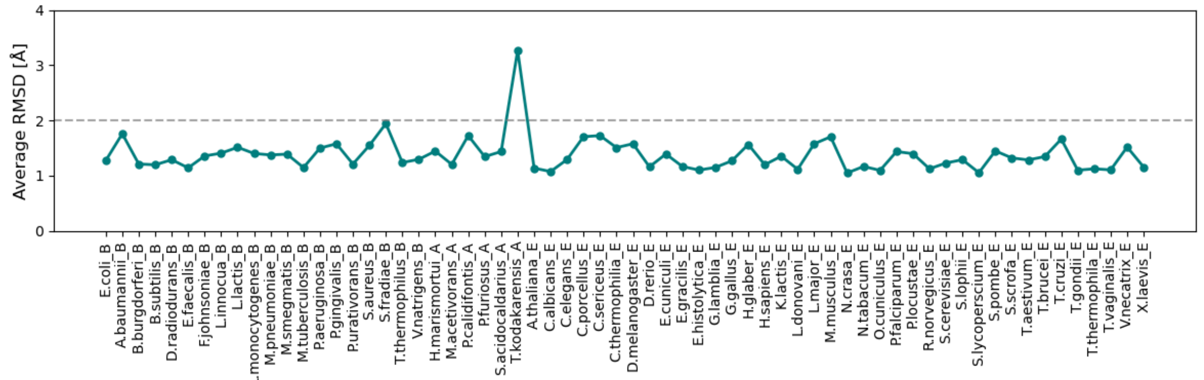


**Supplementary Figure S1. Structural conservation of the Peptidyl Transferase Centre (PTC) across phylogeny. (A)** Pairwise root-mean-square deviation (RMSD) matrix for the PTC region calculated across 64 ribosome structures representing Bacteria, Archaea, and Eukarya. **(B)** Hierarchical clustering dendrogram derived from the pairwise RMSD matrix using the average linkage method, which broadly recapitulates the separation between domains. **(C)** Average RMSD values for each of the 64 PTC structures, highlighting the high degree of structural conservation across the dataset with limited outliers (e.g., *T. kodakarensis*).

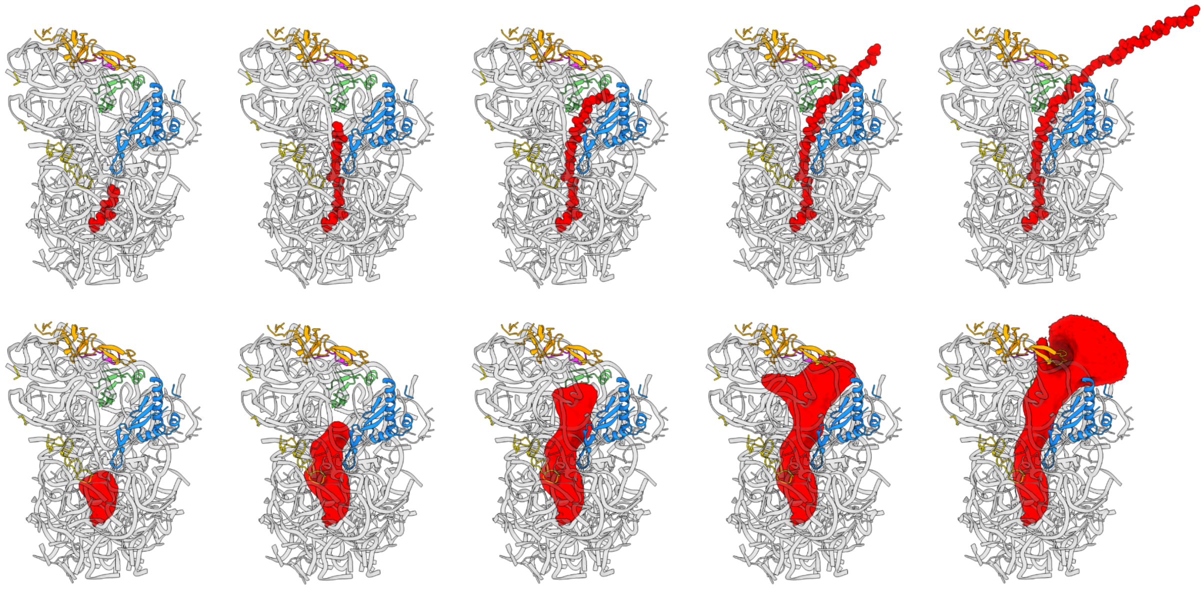

**Supplementary Figure S2. Generation of functional tunnel maps using a nascent chain-centric approach. Top:** Initial structural models of RNC complexes used for simulations with polyalanine nascent chains of increasing lengths (10, 20, 30, 40, and 60 residues) were attached to the P-site tRNA to probe different biosynthetic time points. **Bottom:** Representative volumetric density maps derived from the all-atom MD simulations. These maps represent the spatial occupancy of the nascent chain, defining the functional volume of the tunnel accessible at a specific chain length.

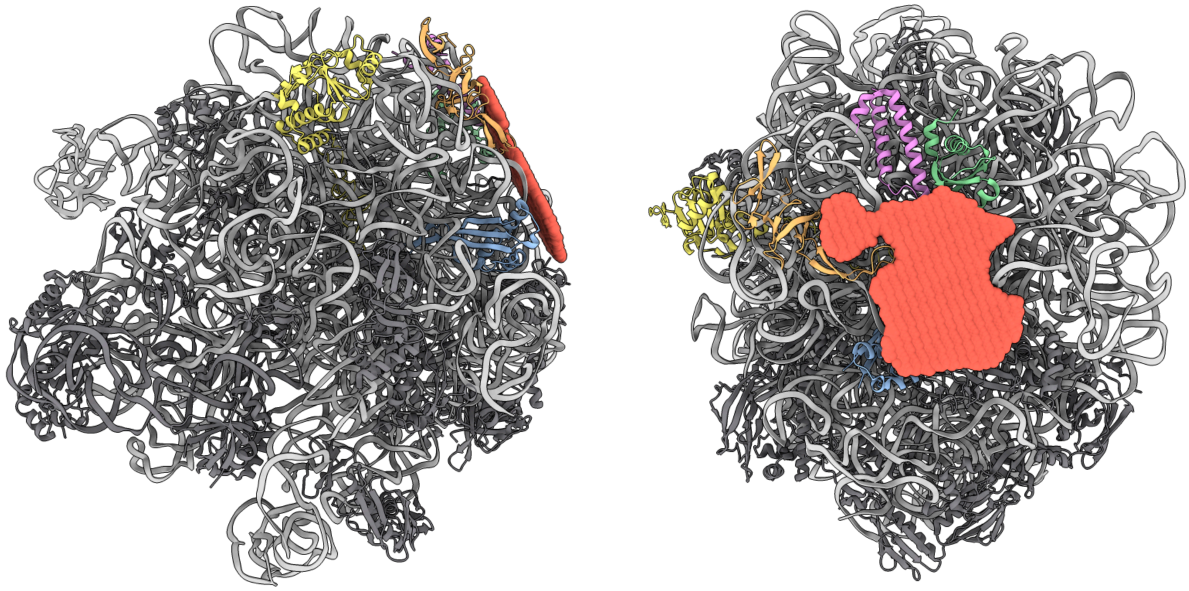

**Supplementary Figure S3. Definition of the ribosomal tunnel exit boundary.** The lower boundary of the exit tunnel was defined by a density plane (red disc) aligned with the conserved loop of protein uL24. Key tunnel-lining proteins are colored for reference: uL4 (yellow), uL22 (blue), uL23 (green), uL24 (orange) and uL29 (violet).

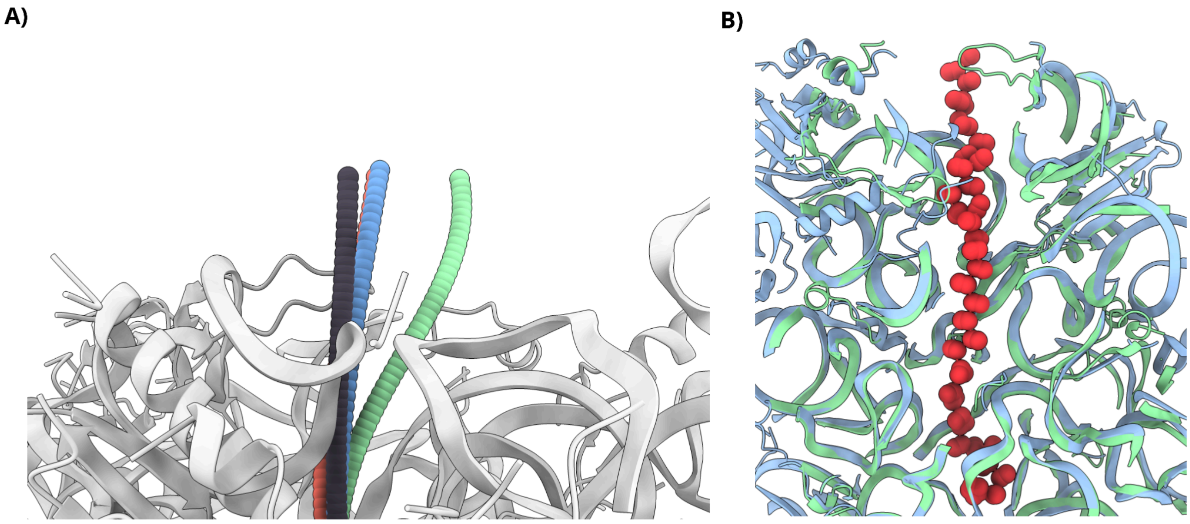


**Supplementary Figure S4. Comparison of nascent chain trajectories and tunnel profiles. (A)** Superposition of nascent chain exit pathways for representative species: *E. coli* (green), *H. sapiens* (blue), *H. marismortui* (red), and *E. cuniculi* (black). **(B)** The cross-section of the ribosome exit tunnel along the main axis for Bacteria (green) and Eukarya (blue), overlaid with a representative nascent chain model (red).

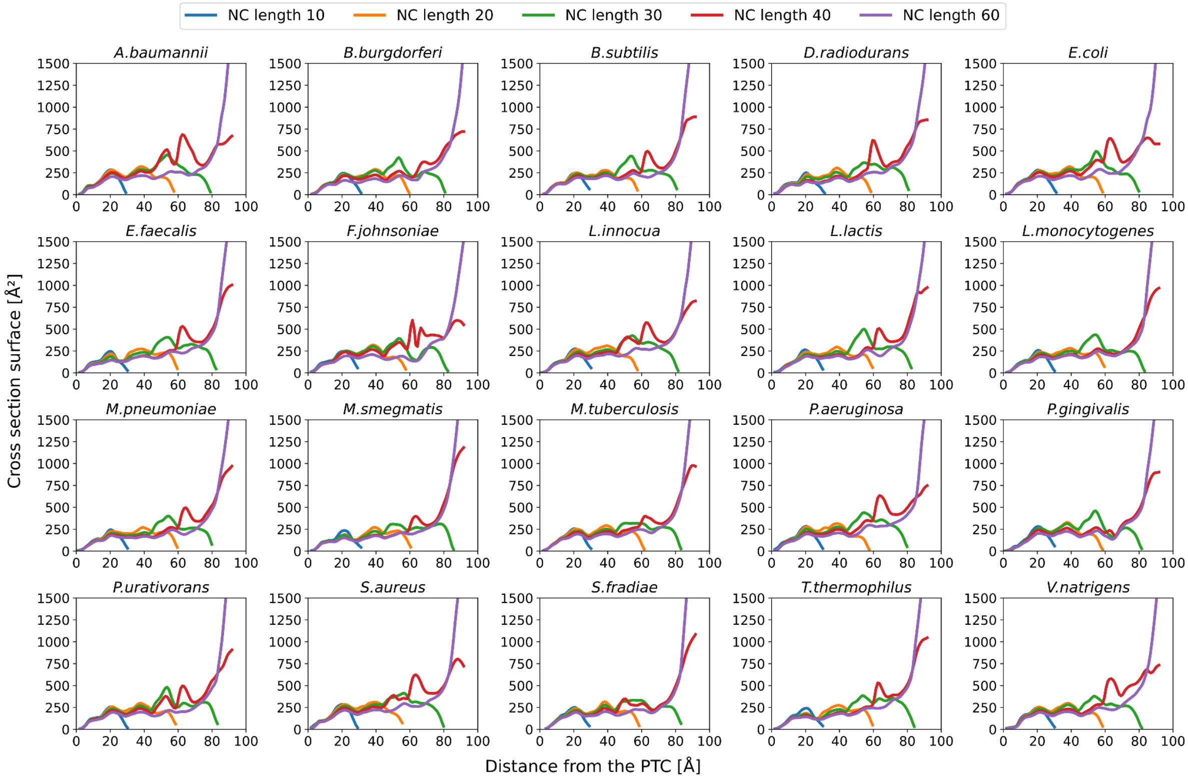


**Supplementary Figure S5. Functional cross-sectional area profiles of bacterial ribosome exit tunnels.** Profiles were calculated for 20 bacterial structures using simulations with five increasing nascent chain lengths (L = 10, 20, 30, 40, and 60 residues), illustrating the length-dependent accessibility of the tunnel volume.

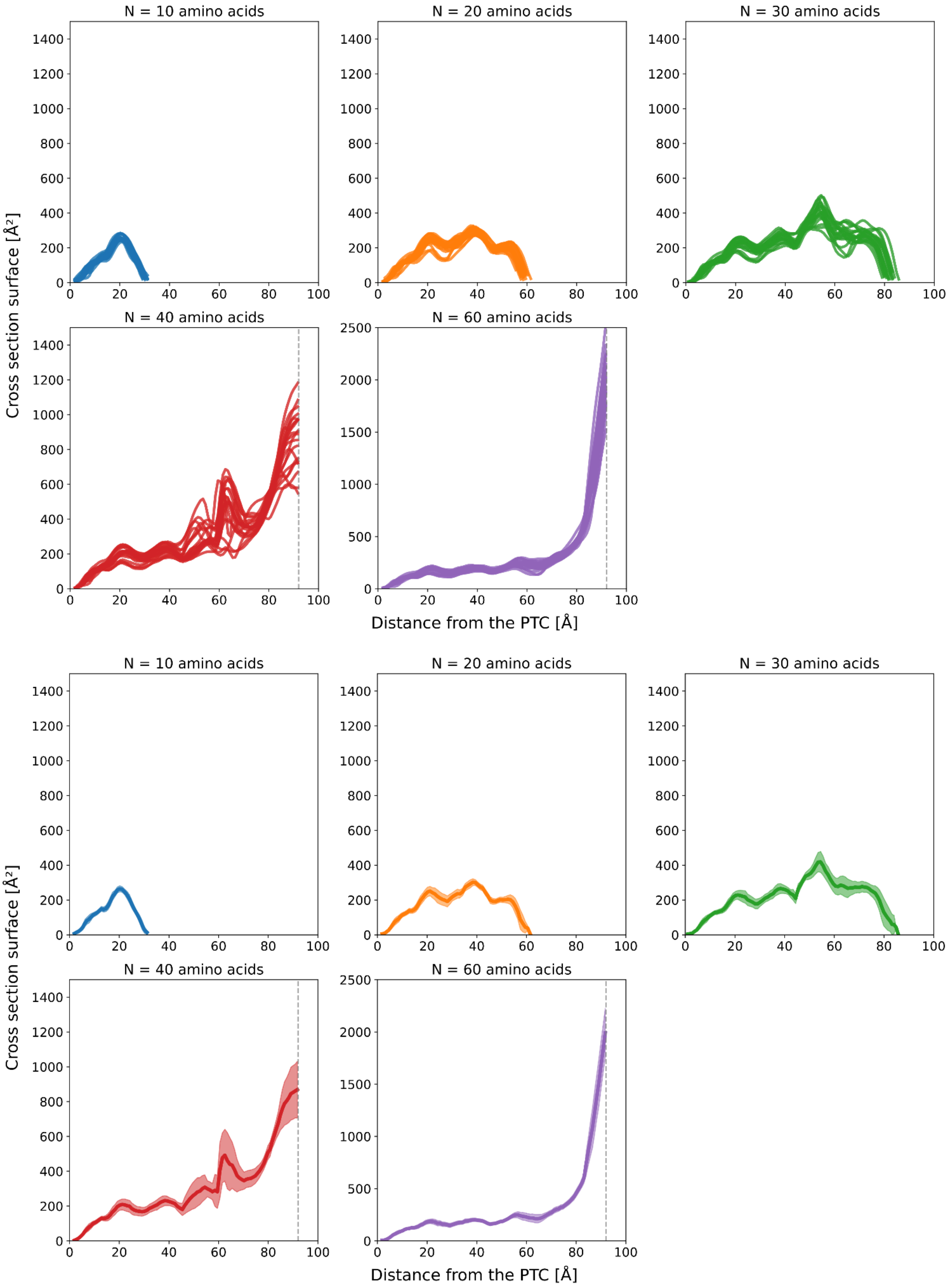


**Supplementary Figure S6. Combined cross-sectional area profiles of bacterial ribosome tunnels. (Top)** Superimposed cross-sectional area profiles derived from all 20 analysed bacterial ribosomes across all nascent chain lengths, visualising the full range of structural variability. **(Bottom)** The average cross-sectional area profile for the bacterial dataset, with the shaded region representing the standard deviation (± SD). A vertical grey dotted line indicates the functional exit boundary of the tunnel.

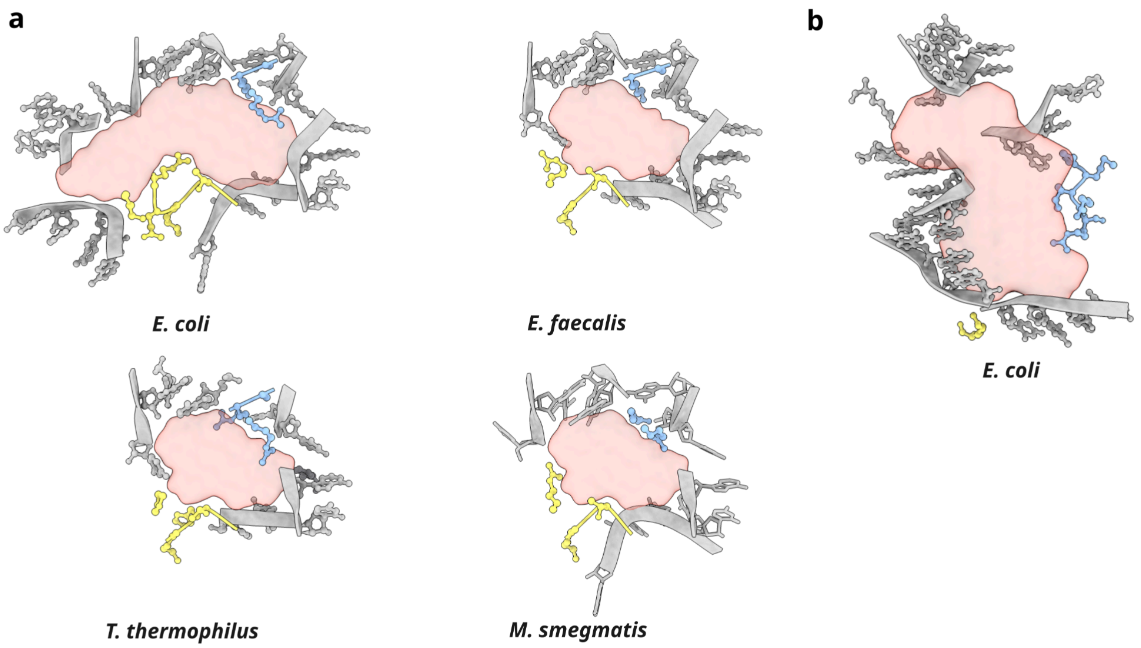


**Supplementary Figure S7. Structural variations at bacterial constriction sites. (A)** Cross-sectional view of the first constriction site (~30 Å from the PTC) in *E. coli*, *E. faecalis*, *T. thermophilus*, and *M. smegmatis*, highlighting species-specific differences in tunnel geometry. **(B)** Structural detail of the second narrowing region in the *E. coli* ribosome. Ribosomal proteins are colored for reference: uL4 (yellow) and uL22 (blue).

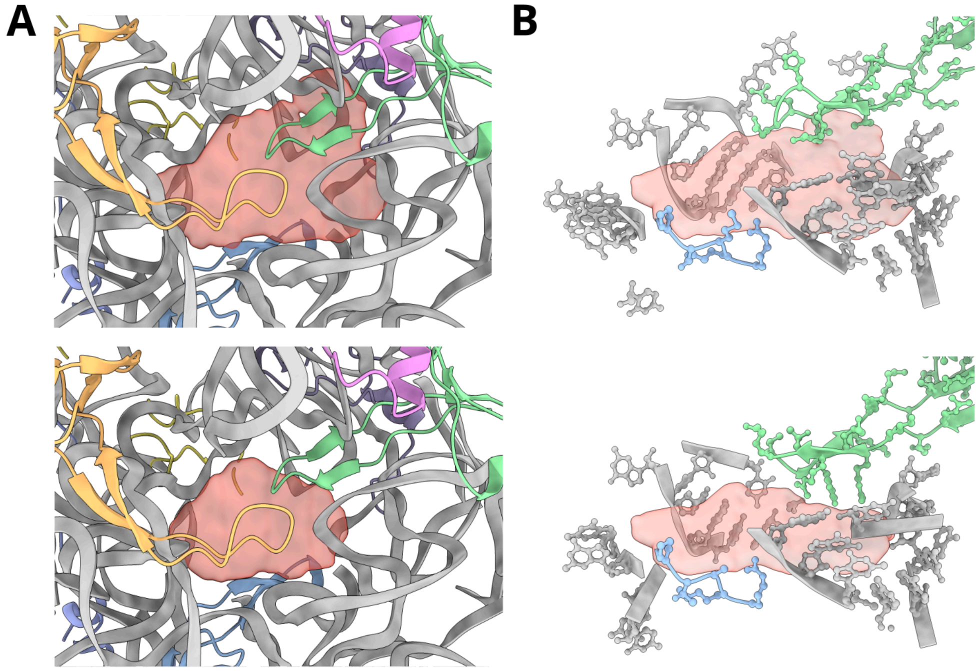


**Supplementary Figure S8. Characterisation of the first bacterial side tunnel and its accessibility. (A)** Cross-sections of the first side tunnel region (~55 Å from the PTC) at nascent chain lengths of L=30 (top) and L=60 (bottom), illustrating the length-dependent gating of this cavity. **(B)** Species-specific structural variation in the first side tunnel (at L=30) between *E. coli* (top) and *M. tuberculosis* (bottom), highlighting local restriction in *M. tuberculosis*. Ribosomal proteins are colored: uL4 (yellow), uL22 (blue), uL23 (green), uL24 (orange), and uL29 (violet).

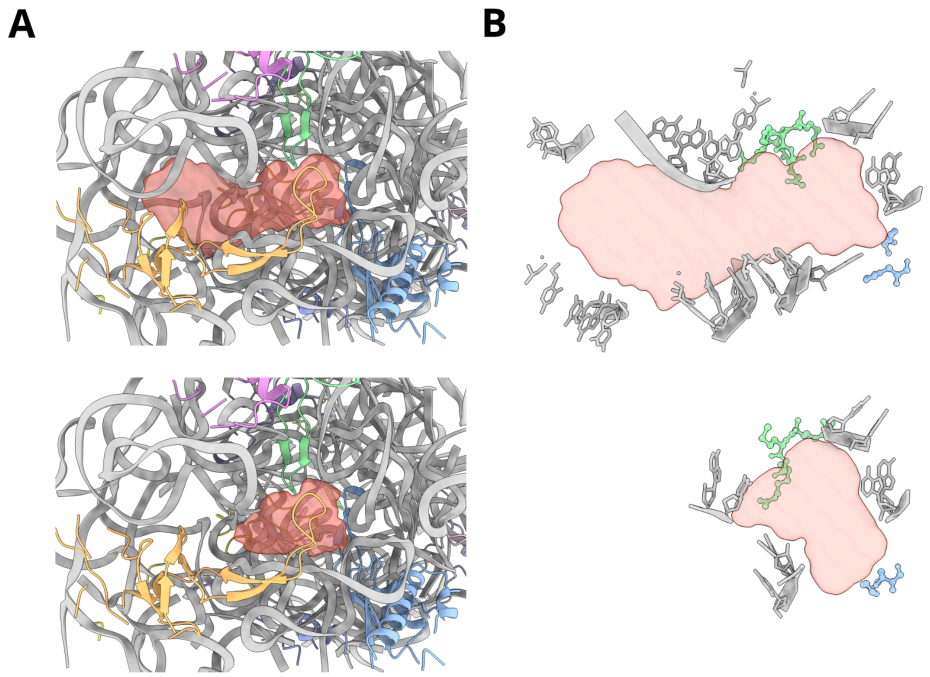


**Supplementary Figure S9. Characterisation of the second bacterial side tunnel. (A)** Cross-sections of the second side tunnel region (~65 Å from the PTC) at nascent chain lengths of L=40 (top) and L=60 (bottom), illustrating changes in accessibility during elongation. **(B)** Structural occlusion of the second side tunnel in *L. monocytogenes*. Comparison of cross-sections at ~65 Å (L=40) between *E. coli* (top, open) and *L. monocytogenes* (bottom, closed). Ribosomal proteins are colored: uL4 (yellow), uL22 (blue), uL23 (green), uL24 (orange), and uL29 (violet).

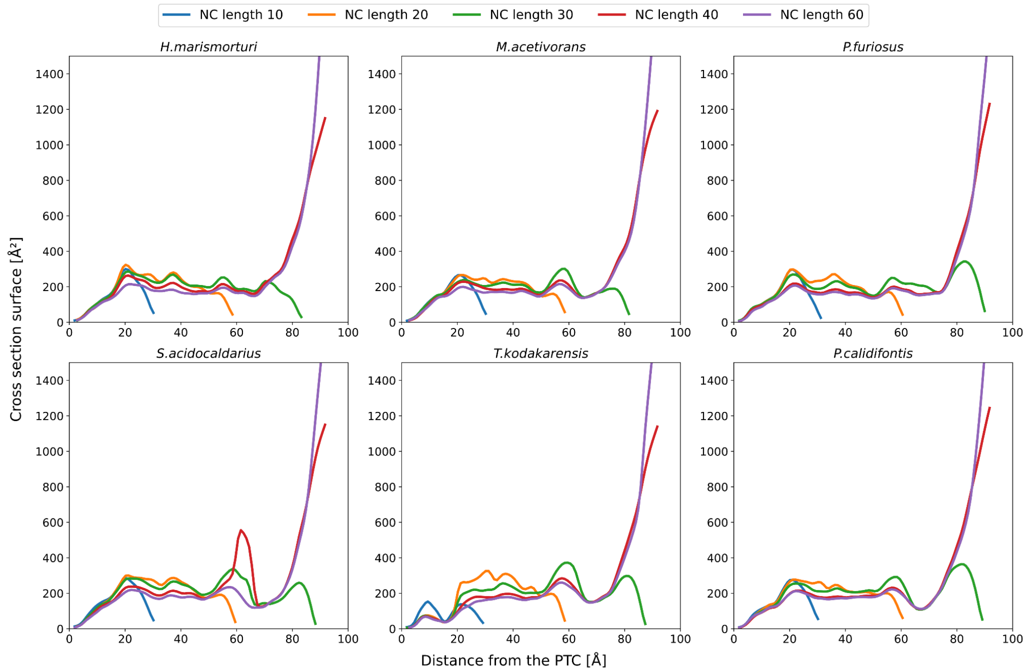


**Supplementary Figure S10. Functional cross-sectional area profiles of archaeal ribosome exit tunnels.** Profiles were calculated for the six available archaeal structures using simulations with five increasing nascent chain lengths (L = 10, 20, 30, 40, and 60 residues), illustrating the length-dependent accessibility of the tunnel volume.

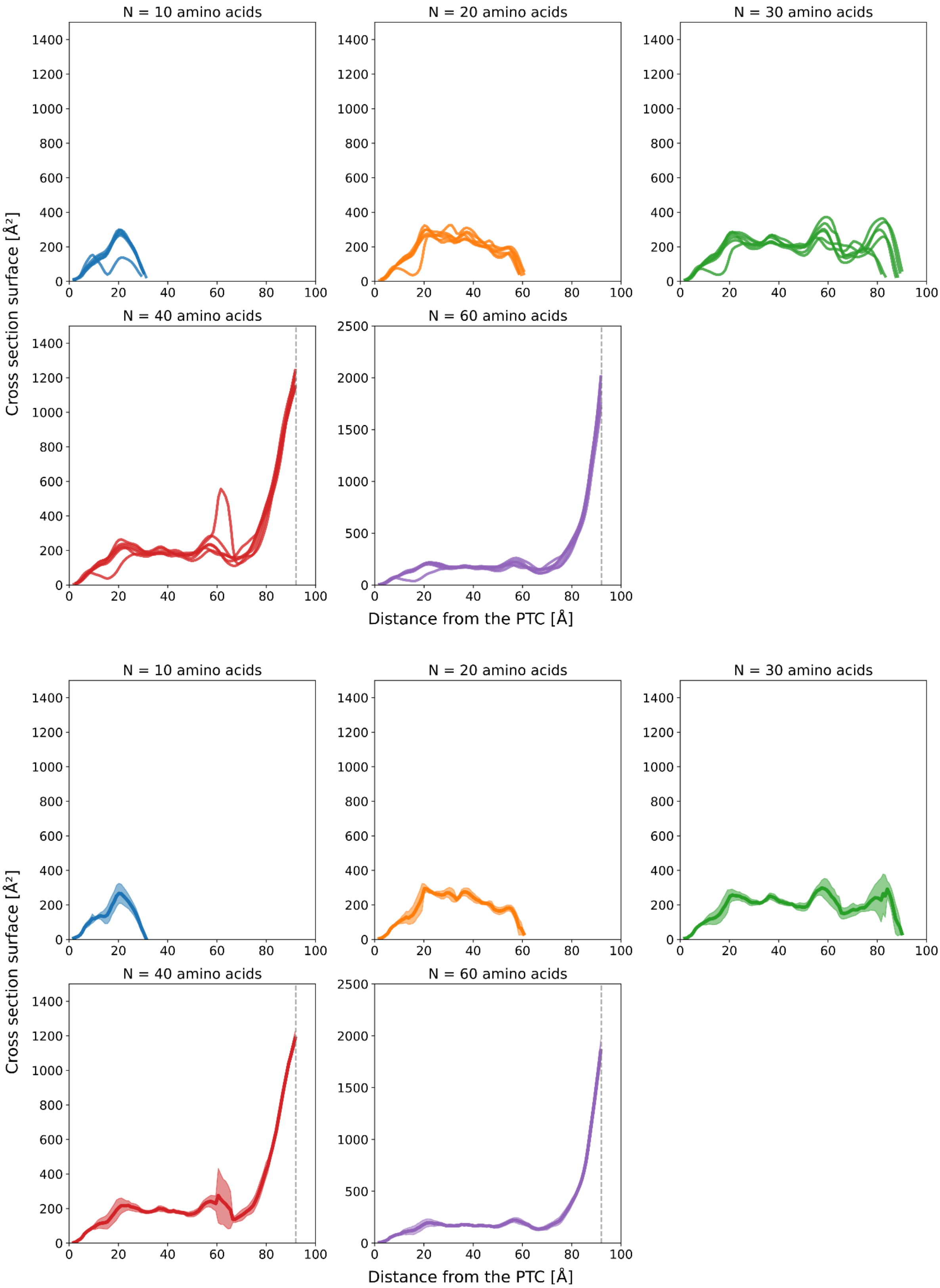


**Supplementary Figure S11. Combined cross-sectional area profiles of archaeal ribosome tunnels. (Top)** Superimposed cross-sectional area profiles derived from all six analysed archaeal ribosomes across all nascent chain lengths, visualising the structural variability within

the domain. **(Bottom)** The average cross-sectional area profile for the archaeal dataset, with the shaded region representing the standard deviation (± SD). A vertical grey dotted line indicates the functional exit boundary of the tunnel.

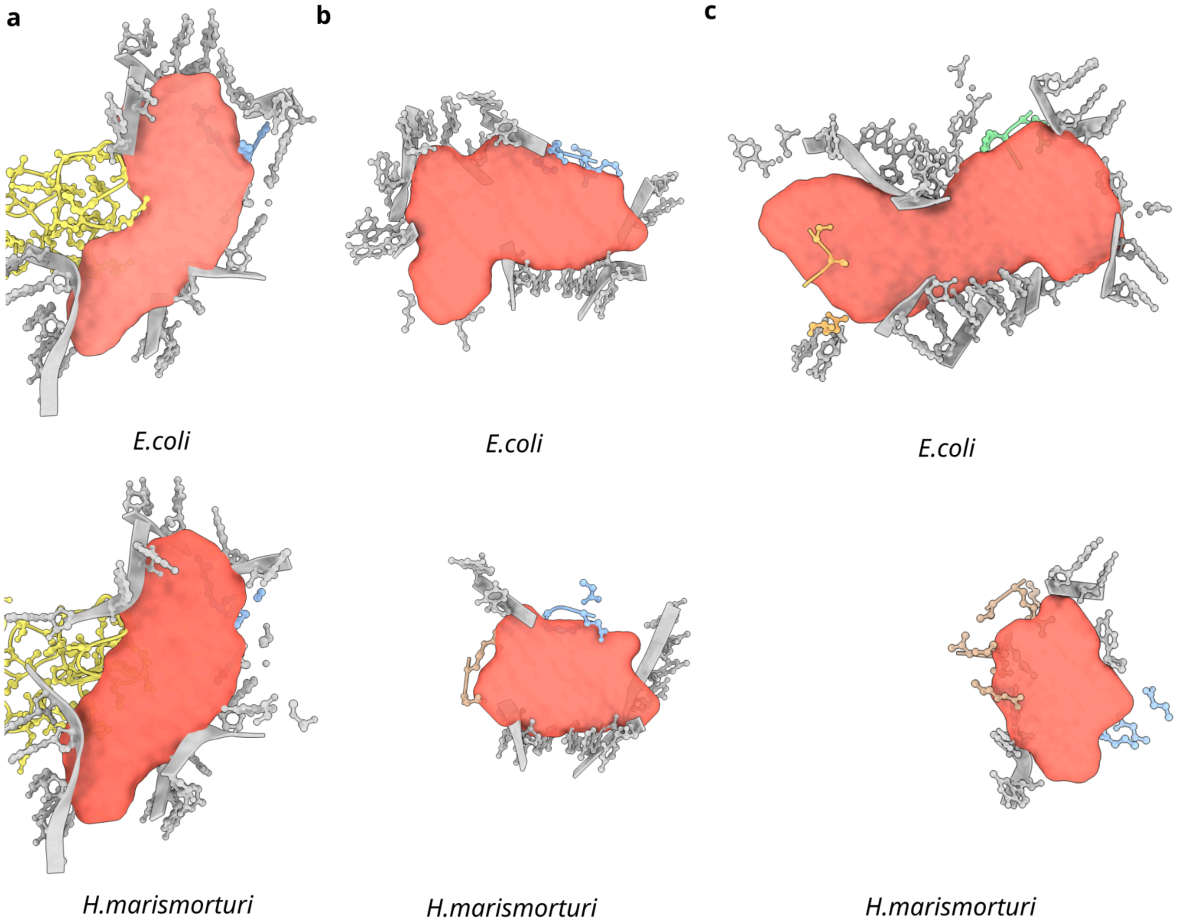


**Supplementary Figure S12. Structural comparison of bacterial openings and archaeal constrictions. (A)** The first constriction site (~30 Å from PTC): Comparison between *E. coli* (top) and *H. marismortui* (bottom), showing the wider architecture in Archaea. **(B)** The second archaeal constriction region (~50 Å): *E. coli* (top) exhibits an open lateral cavity (first side tunnel), whereas *H. marismortui* (bottom) forms a constriction due to the presence of eL39 (light brown) and the uL4 loop (yellow). **(C)** The third archaeal constriction region (~67 Å): *E. coli* (top) shows the opening of the second side tunnel, while *H. marismortui* (bottom) is sealed by eL39. Ribosomal proteins are colored: uL4 (yellow), uL22 (blue), uL23 (green), uL24 (orange), and eL39 (light brown).

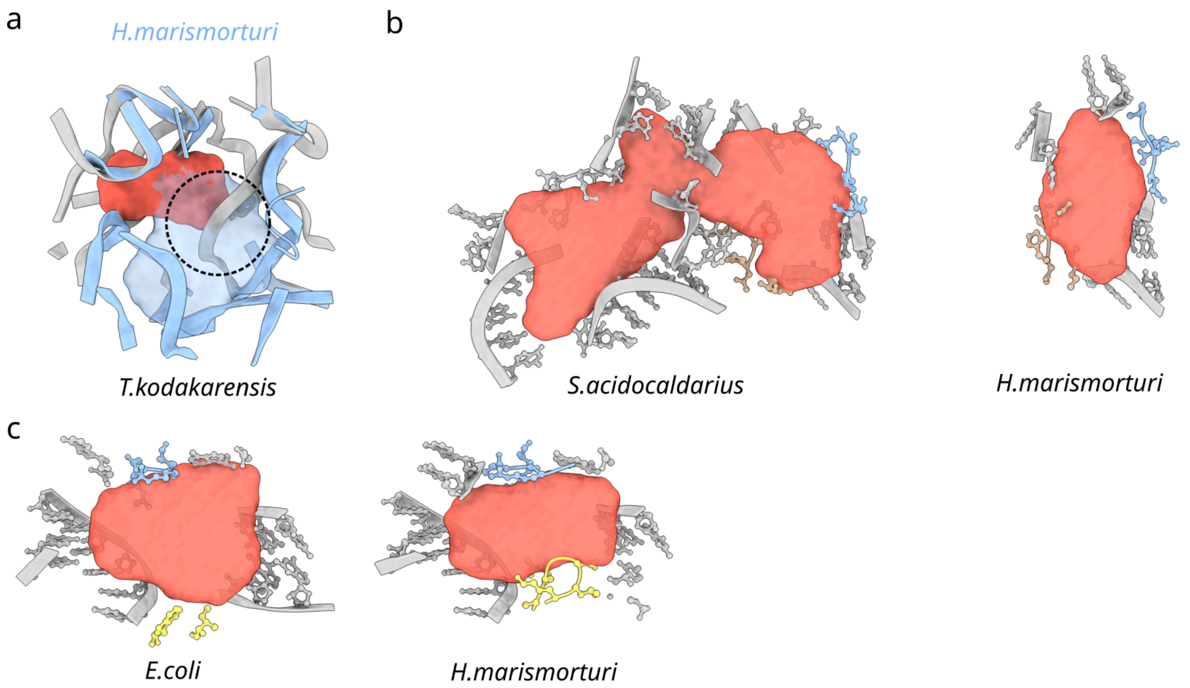


**Supplementary Figure S13. Structural anomalies and adaptations in archaeal tunnels. (A)** Unique PTC obstruction in *T. kodakarensis*. Comparison of the PTC region in *H. marismortui* (left) and *T. kodakarensis* (right). The circled region highlights a distinct rRNA conformation in *T. kodakarensis* that sterically occludes the nascent chain path. **(B)** Atypical side tunnel retention in *S. acidocaldarius*. Cross-sections showing the "side tunnel" region in *S. acidocaldarius* (left, open) compared to the canonical "sealed" architecture of *H. marismortui* (right). **(C)** Modulation of tunnel geometry by the second uL4 loop. Visualisation of how the archaeal-specific second loop of uL4 (yellow) remodels the tunnel cross-section, contributing to increased asphericity. Ribosomal proteins are colored: uL4 (yellow) and uL22 (blue).

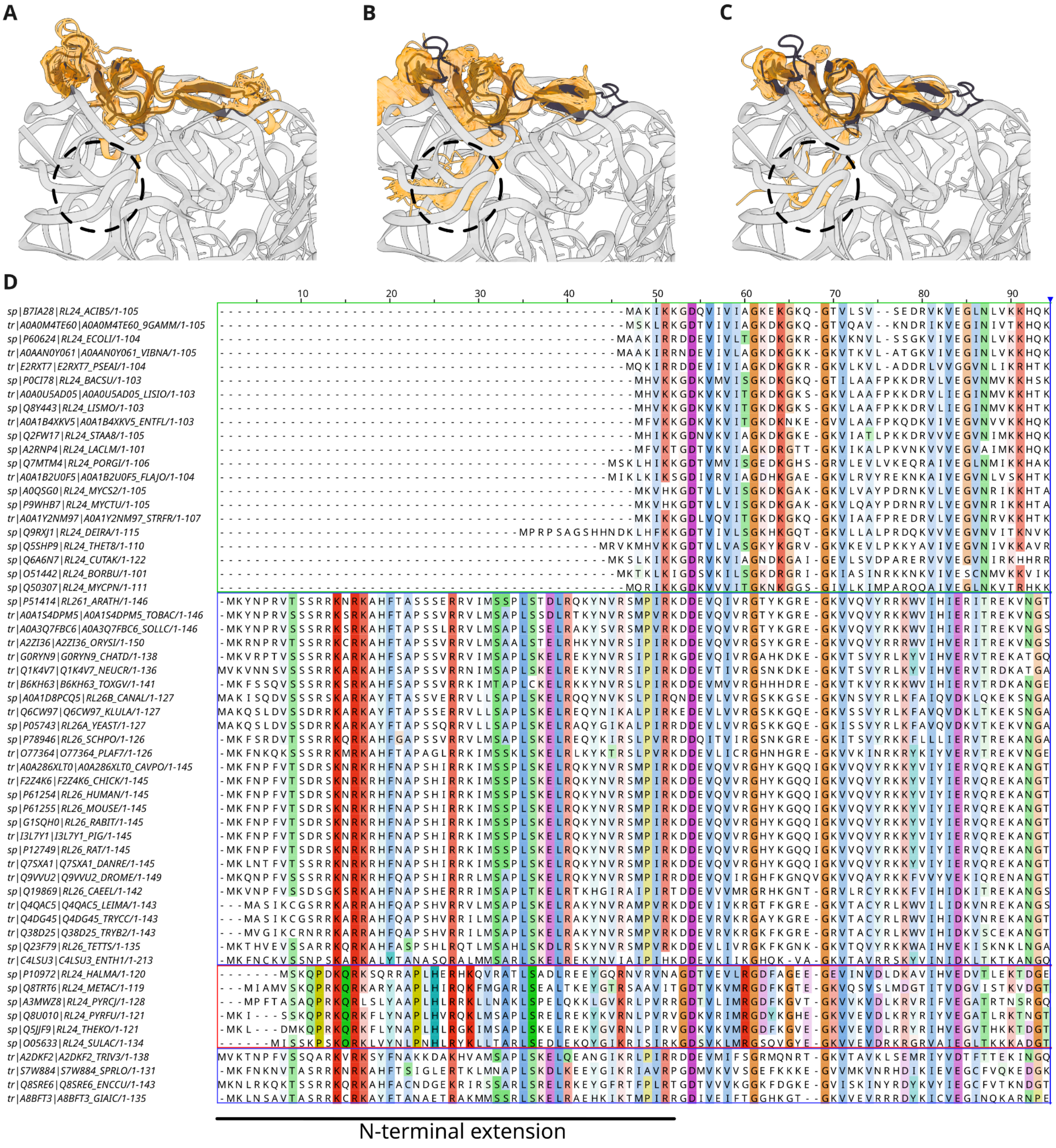


**Supplementary Figure S14. Evolutionary adaptation of the uL24 protein seals the bacterial-specific lateral branch in the eukaryotic and archaeal exit tunnel vestibule. (A)** Structural ensemble of uL24 from various bacterial ribosomes (orange) superimposed onto the *E. coli* reference structure (uL24 in black, rRNA and other ribosome proteins in light grey), demonstrating the absence of the N-terminal extension in the highlighted region (black dotted circle). **(B–C)** Structural ensembles of uL24 from various **(B)** eukaryotic and **(C)** archaeal ribosomes (orange) superimposed onto the *E. coli* reference. Both domains possess a highly conserved N-terminal extension that physically occupies the volume corresponding to the bacterial distal lateral branch (black dotted circle; see also Fig. 3c). **(D)** Multiple sequence alignment of uL24 proteins confirming the conservation of this N-terminal extension in Eukaryotes (blue box) and Archaea (red box) compared to Bacteria (green box), highlighting a major lineage-specific adaptation in the co-translational folding environment.

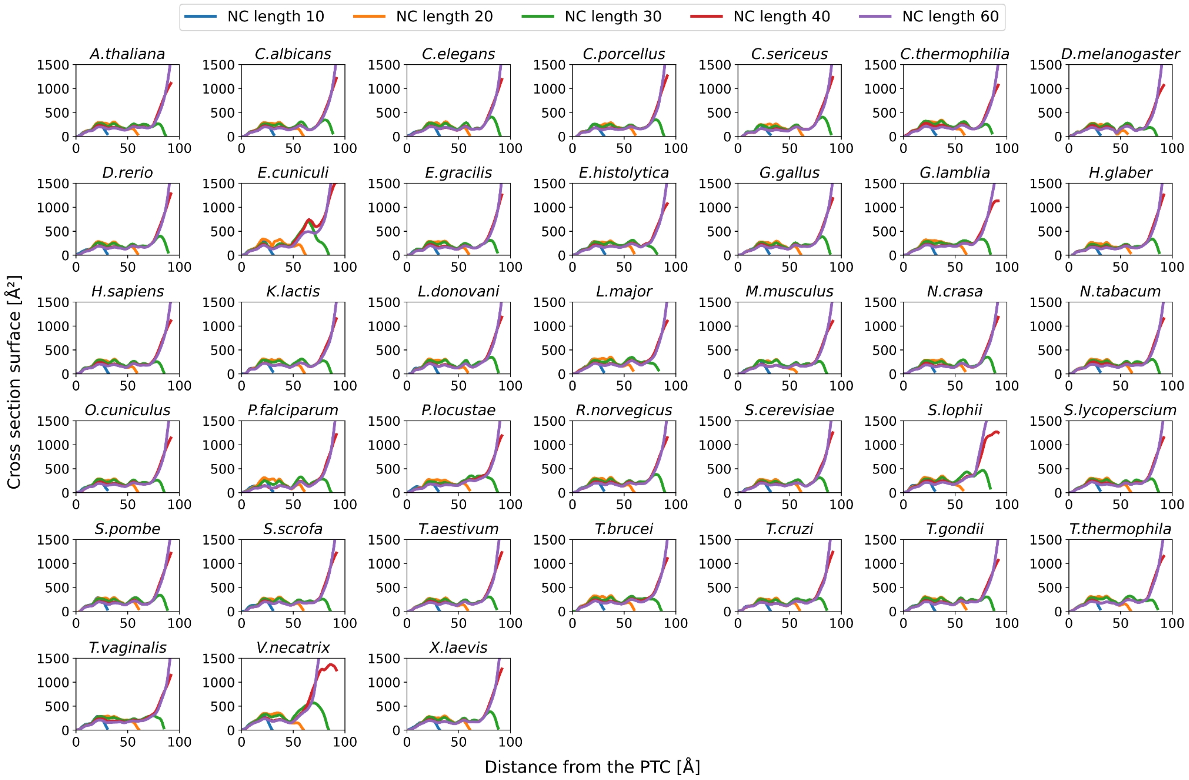


**Supplementary Figure S15. Functional cross-sectional area profiles of eukaryotic ribosome exit tunnels.** Profiles were calculated for 38 eukaryotic structures using simulations with five increasing nascent chain lengths (L = 10, 20, 30, 40, and 60 residues), illustrating the length-dependent accessibility of the tunnel volume.

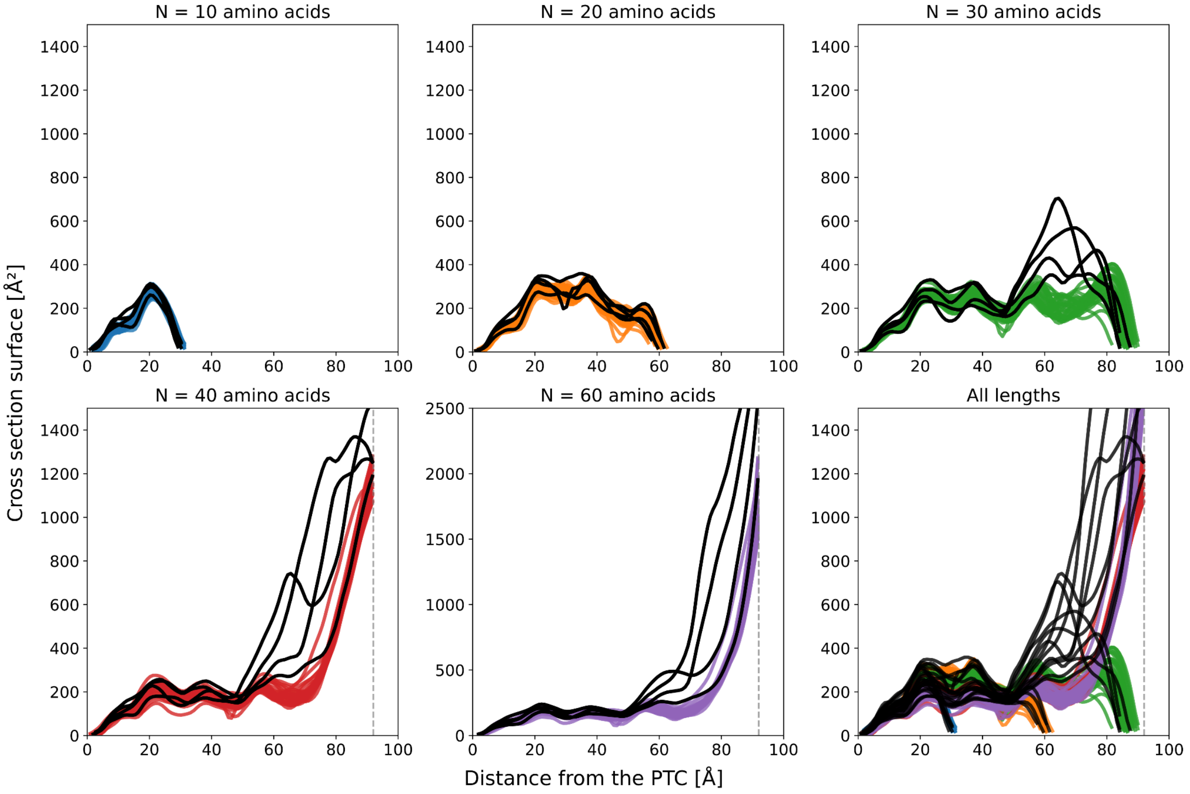

**Supplementary Figure S16. Combined cross-sectional area profiles of eukaryotic ribosome tunnels.** Superimposed cross-sectional area profiles derived from all 38 eukaryotic ribosomes. Profiles corresponding to Microsporidia species are highlighted in black, illustrating their divergent tunnel architecture compared to canonical eukaryotes. The functional exit boundary is indicated by a vertical grey dotted line.

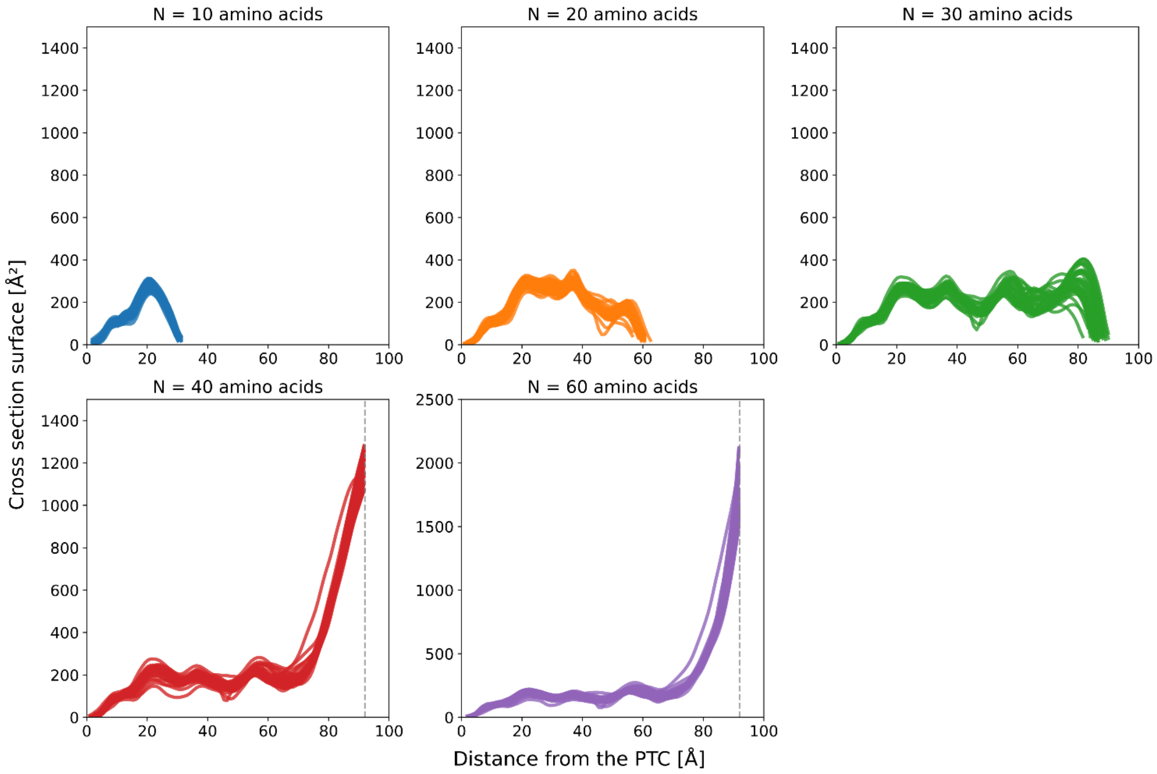


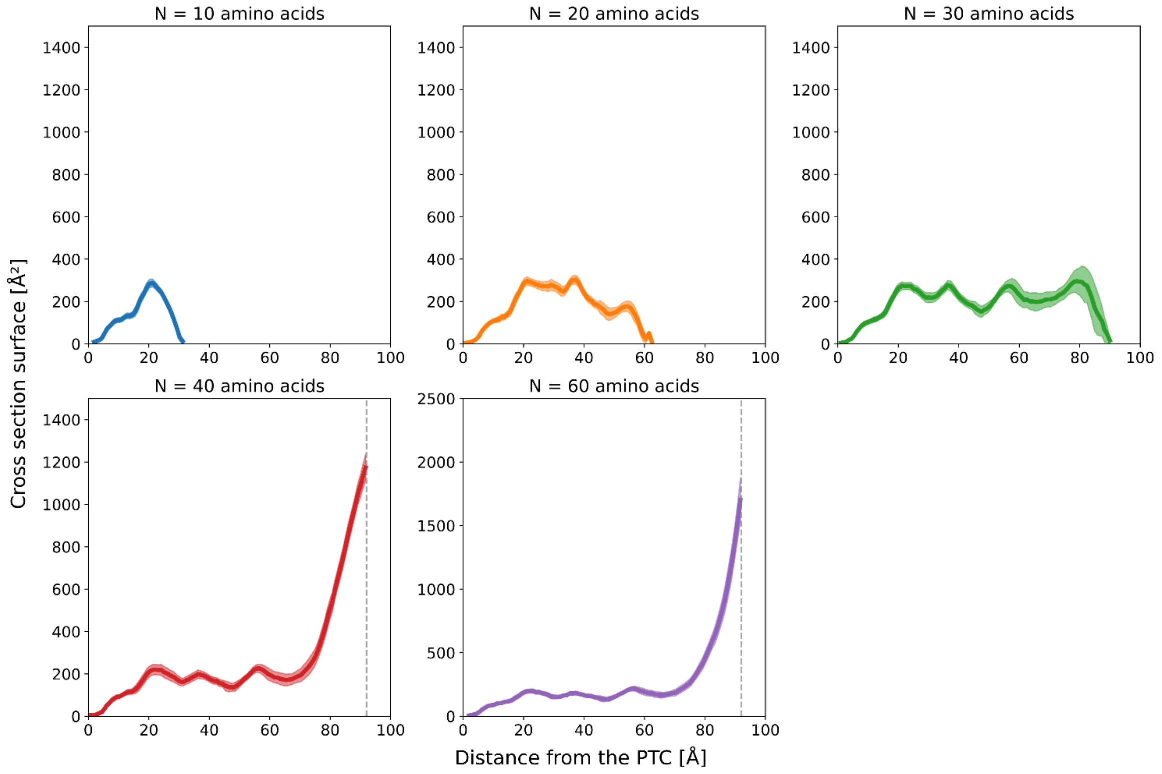

**Supplementary Figure S17. Combined cross-sectional area profiles of canonical eukaryotic ribosome tunnels. (Top)** Superimposed cross-sectional area profiles derived from eukaryotic ribosomes, excluding Microsporidia structures, to visualise the structural conservation of the canonical eukaryotic tunnel. **(Bottom)** The average cross-sectional area profile for the canonical eukaryotic dataset, with the shaded region representing the standard deviation (± SD). A vertical grey dotted line indicates the functional exit boundary of the tunnel.

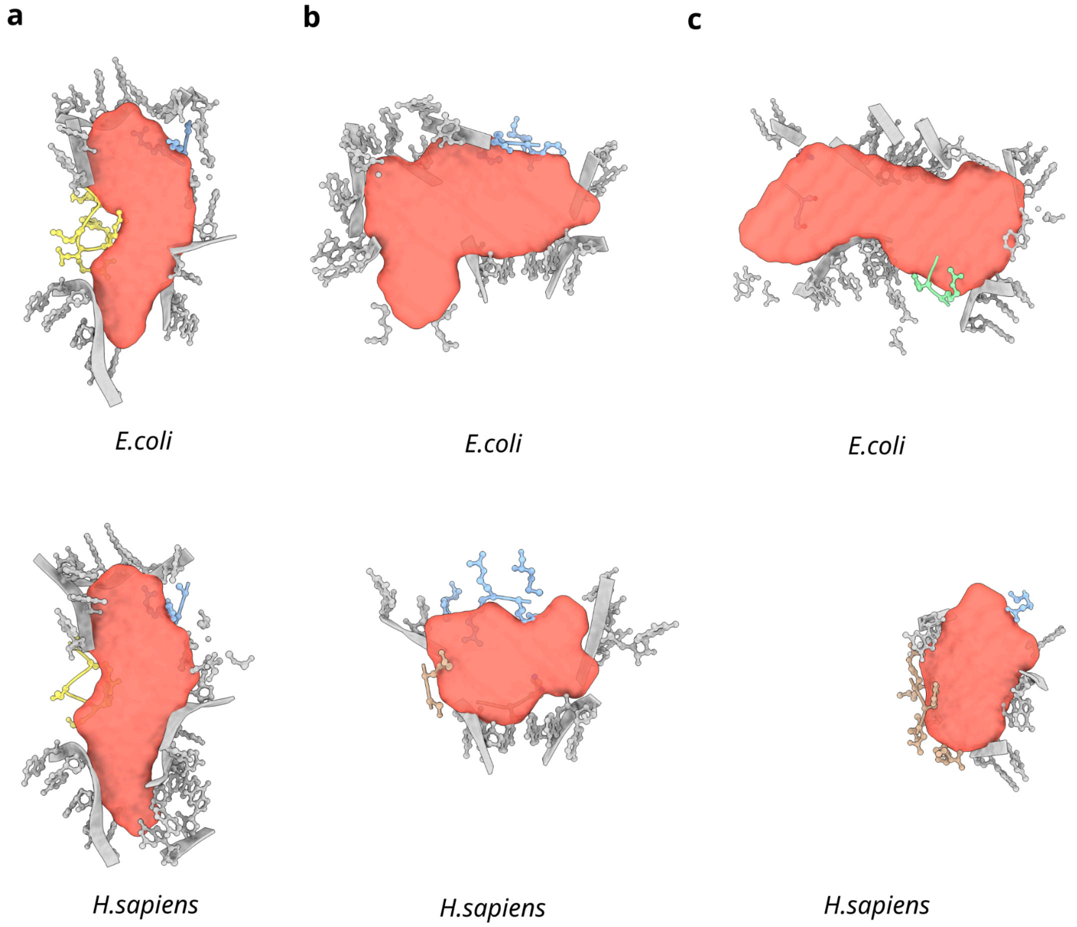


**Supplementary Figure S18. Structural closure of ancestral side tunnels in the eukaryotic ribosome. (A)** Comparison of the first constriction site in *E. coli* (top) and *H. sapiens* (bottom). **(B)** The first side tunnel region: *E. coli* (top) displays an accessible lateral cavity, whereas the corresponding region in *H. sapiens* (bottom) is structurally occluded. **(C)** The second side tunnel region: The open cavity found in *E. coli* (top) is physically sealed in *H. sapiens* (bottom) by the eukaryotic-specific protein eL39 (light brown). Ribosomal proteins are colored: uL4 (yellow), uL22 (blue), uL23 (green), and eL39 (light brown).

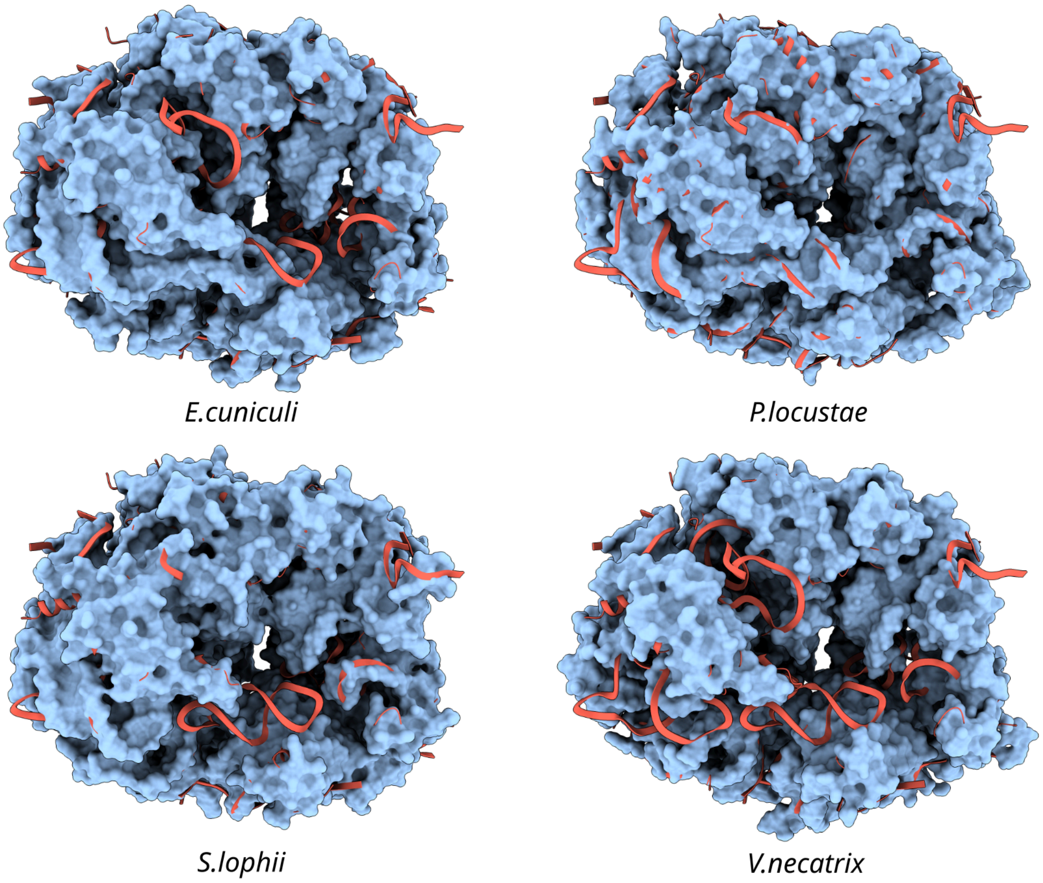


**Supplementary Figure S19. Structural divergence of the Microsporidia exit tunnel.** Superposition of the Microsporidia exit tunnel volume (blue surface) onto the *H. sapiens* ribosome structure (red cartoon), viewed from the top. This comparison illustrates the distinctive reduced geometry of the parasitic tunnel relative to the canonical human architecture.

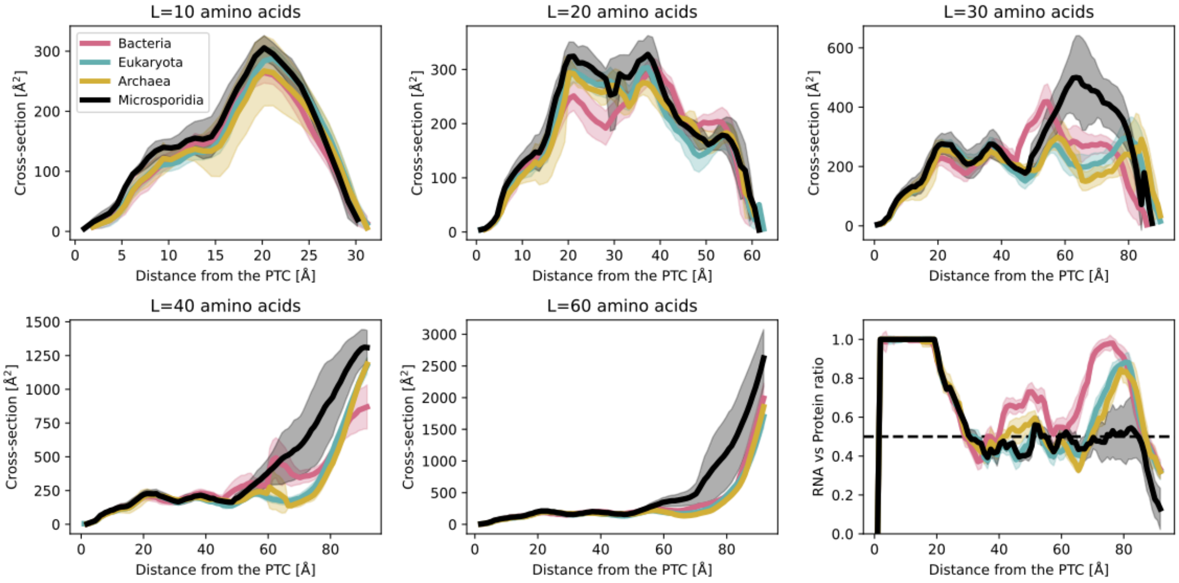


**Supplementary Figure S20. Global comparison of ribosome tunnel functional profiles.**
Superposition of the average cross-sectional area profiles for Bacteria (red), Archaea (orange),

canonical Eukaryotes (cyan), and Microsporidia (black). The shaded regions represent the standard deviation (± SD) for each group. This comparison illustrates the distinct geometric divergence between the domains.
